# Tumor-resident microbiota contributes to colorectal cancer liver metastasis by lactylation and immune modulation

**DOI:** 10.1038/s41388-024-03080-7

**Published:** 2024-06-18

**Authors:** Jian Gu, Xiaozhang Xu, Xiangyu Li, Lei Yue, Xiaowen Zhu, Qiuyang Chen, Ji Gao, Maruyama Takashi, Wenhu Zhao, Bo Zhao, Yue Zhang, Minjie Lin, Jinren Zhou, Yuan Liang, Shipeng Dai, Yufeng Pan, Qing Shao, Yu Li, Yiming Wang, Zibo Xu, Qufei Qian, Tianning Huang, Xiaofeng Qian, Ling Lu

**Affiliations:** 1 Hepatobiliary Center, The First Affiliated Hospital of Nanjing Medical University and Research Unit of Liver Transplantation and Transplant Immunology, Chinese Academy of Medical Sciences, Nanjing, China; 2 Jiangsu Key Laboratory of Cancer Biomarkers, Prevention and Treatment, Collaborative Innovation Center for Cancer Personalized Medicine, Nanjing Medical University, Nanjing, China; 3 Department of General Surgery, The Affiliated BenQ Hospital of Nanjing Medical University, Nanjing, China; 4 Zhejiang Cancer Hospital, Hangzhou Institute of Medicine (HIM), Chinese Academy of Sciences, Hangzhou, China; 5 National Institutes of Health (NIH), New York, NY USA; 6 National and Local Joint Engineering Research Center of Biomedical Functional Materials, School of Chemistry and Materials Science, Nanjing Normal University, Nanjing, China; 7 The Clinical Skills Training Center, The Second Xiangya Hospital of Central South University, Changsha, China; 8 School of Biological Science & Medical Engineering, Southeast University, Nanjing, China; 9 School of Medicine, Southeast University, Nanjing, China

**Keywords:** Cancer immunotherapy, Diagnostic markers

## Abstract

The role of tumor-resident microbiota in modulating tumor immunity remains unclear. Here, we discovered an abundance of intra-tumoral bacteria, such us *E.coli*, residing and resulting in Colorectal cancer liver metastasis (CRLM). *E.coli* enhanced lactate production, which mediated M2 macrophage polarization by suppressing nuclear factor-κB -gene binding (NF-κB) signaling through retinoic acid-inducible gene 1 (RIG-I) lactylation. Lactylation of RIG-I suppressed recruitment of NF-κB to the Nlrp3 promoter in macrophages, thereby reducing its transcription. This loss of Nlrp3 affected the immunosuppressive activities of regulatory T cells (Tregs) and the antitumor activities of and CD8^+^ T cells. Small-molecule compound screening identified a RIG-I lactylation inhibitor that suppressed M2 polarization and sensitized CRLM to 5-fluorouracil (5-FU). Our findings suggest that tumor-resident microbiota may be a potential target for preventing and treating CRLM.

## Introduction

Colorectal cancer (CRC) is one of the most common malignancies worldwide and the second leading cause of cancer-related death. CRC also has a high rate of recurrence and metastasis, despite advances in treatment [1]. Colorectal cancer liver metastasis (CRLM) is the most common distant metastasis of CRC and causes a high proportion of cancer-related deaths [2]. Besides the anatomical structure relationship between the liver and colorectum, the immune microenvironment of the liver is highly immunosuppressive, which can dampen the antitumor immune response, making the liver an attractive metastatic site [3]. As an important part of the liver tumor microenvironment, immune cells play a key role in the formation and prevention of tumors [4, 5]. However, how CRLM affects immune cells within the tumor microenvironment (TME) and the underlying mechanisms of CRLM progression are largely unknown.

Tumor-resident microbiota may affect tumorigenesis and progression. Microbiota inside the tumor [6] modulates tumor microenvironment (TME) by inducing gene mutations [7], regulating oncogenic pathways [8], and modulating the host immune system [9]. The microbiota in the gut promotes CRC progression by suppressing local immune activity [10]; the gut microbiota can also promote the intestinal dissemination of cancer cells, leading to metastasis [11]. However, the impact and immune regulation mechanism of tumor-resident microbiota on distant metastatic organs has rarely been described.

Given that primary CRC and liver metastasis are colonized by similar bacteria [12, 13], we hypothesized that tumor-resident microbiota regulates the TME and facilitate tumor planting and progression in the liver. Thus, we aimed to investigate the specific mechanism by which tumor-resident microbiota promotes CRLM by regulating the immune microenvironment of the liver and to design and apply potential novel anti-microbiota-based therapy combined with chemo or immune therapy for CRLM patients.

## Results

### CRLM has significant amounts of microbiota, which promote disease progression

**Fig. 1:**
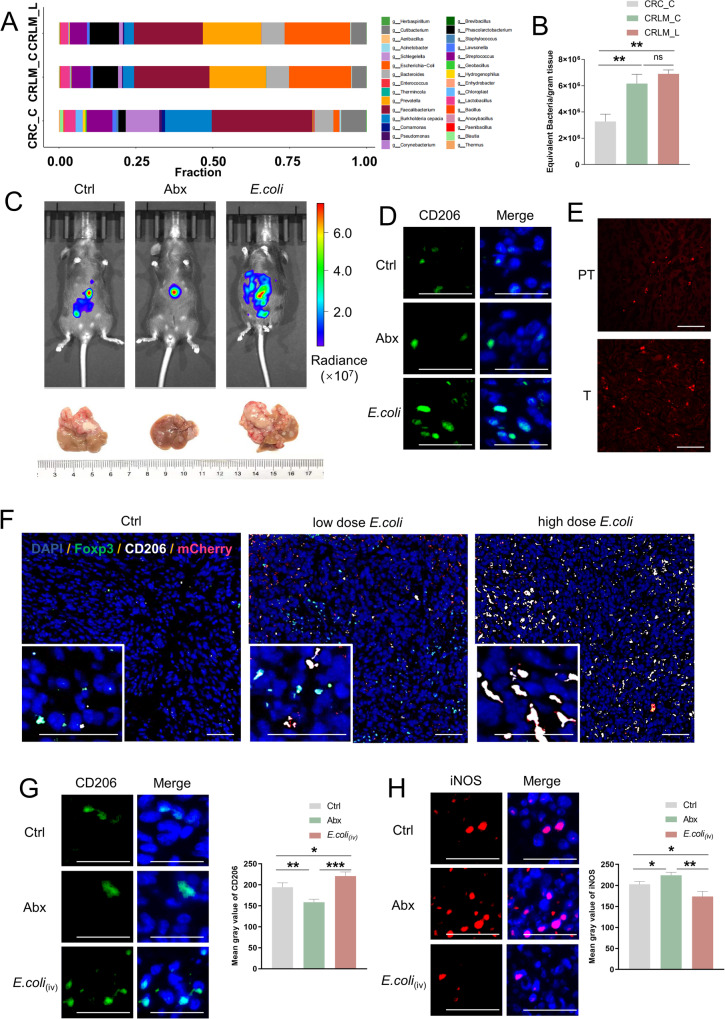
CRLM has significant amounts of microbiota, which promotes disease progression. **A** Overview of the fraction of microbiota from the tumor samples by 16 s rDNA sequencing. Patients are grouped into separate plots based on sample location and with or without liver metastasis (CRC-C, CRLM-C, and CRLM-L). Each color represents a kind of microbiota, and the length of each color represents the abundance of the microbiota in each kind of tumor sample in general. **B** The numbers of microbiota from the tumors (*n* = 20) in CRC-C, CRLM-C, and CRLM-L were collected and detected by qPCR. Error bars indicate SEM. Statistics were determined using a *t*-test with significance indicated (ns, not significant. **p < 0.01). **C** Representative whole-body bioluminescence images (up) of mice orthotopically xenografted after intravenous injection with MC38-luc+ cells and representative images of liver metastasis (down) from control, antibiotics-treated group (Abx) and *E.coli*-treated group mice (*n* = 10). **D** Representative co-immunofluorescence images of CD206(M2 marker) and DAPI (nuclear counterstain) in tumors from mice in (**C**). Scale bars, 100 μm. Objective, 10x. **E** Representative immunofluorescence images of mCherry(*E.coli* marker) in para tumor (PT) and liver tumor (T) from mice gavaged with mCherry-*E.coli* at day 21. Scale bars, 100 μm. Objective, 5x. **F** Representative co-immunofluorescence images of staining for mCherry(*E.coli* marker), CD206(M2 marker), Foxp3(Treg marker), and DAPI(nuclear counterstain) in tumors from mice in liver metastasis from control, low dose and high dose *E.coli*-treated group mice (*n* = 10) at day 21. Scale bars, 100 μm Objective, 10x and 5x. **G** Representative co-immunofluorescence images of staining for CD206(M2 marker) and DAPI (nuclear counterstain) liver metastasis from control, antibiotics-treated, and *E.coli*(iv) injected group mice (*n* = 10) at day 21. Scale bars, 100 μm. Objective, 10x. **H** Representative co-immunofluorescence images of staining for iNOS(M1 marker) and DAPI(nuclear counterstain) liver metastasis from control, antibiotics-treated, and *E.coli*(iv) injected group mice (*n* = 10) at day 21. Scale bars, 100 μm. Objective, 10x.

To clarify the correlation between microbiota and CRLM, we sequenced the bacterial 16 S rDNA from resected tumor tissues of twenty-four Chinese patients with non-metastatic CRC (CRC-C) and twenty Chinese patients with metastatic disease [primary tumor tissues (CRLM-C) and liver metastatic tissues (CRLM-L)] (Supplementary Fig.1A; Supplementary Table S1). Total genomic DNA was extracted from tumor tissues using the QIAamp PowerFecal (pro) DNA kit (QIAGEN-#51804, Dusseldorf, Germany) according to the manufacturer’s instructions. We ensured consistency and reliability by subjecting negative controls, including water and untreated samples, to the same DNA extraction and PCR procedures as experimental groups. Owing to the low biomass of intra-tumoral microbiota, we used an optimized Real-time Quantitative PCR (qPCR) Detecting System protocol [14] and achieved a detection sensitivity of 5 × 10^3^ equivalent bacteria per gram of tissue. The compositions of the microbiota in each group are shown in Fig.1B. CRLM-C and CRLM-L samples had more diverse and richer microbiota than CRC-C samples (Fig. 1A, B). We detected 6.1 × 10^6^ and 6.8 × 10^6^ equivalent bacteria per gram of tissue in CRLM-C and CRLM-L samples, respectively, whereas only 3.3 × 10^6^ equivalent bacteria per gram of tissue were detected in CRC-C samples (Fig. 1B). The bacterial load and diversity were similar in CRLM-C and CRLM-L tissues (Fig. 1A, B, Supplementary Fig.1A–C), which is consistent with the results of Bullman et al. [15] Further, Many bacteria are specifically highly enriched in CRLM, such as *Prevotella*, *E.coli* and *Phascolarctobacterium* (Supplementary Fig. 1B, D). Besides, compared with that in CRLM-L samples, the abundance of equivalent bacteria in the liver tissues of patients with its para-cancerous tissue (CRLM-L-PT) was significantly reduced, whereas there was no apparent difference between CRC-C samples and its para-cancerous tissue (CRC-PT) in the no liver metastasis patients (Supplementary Fig. 1E).

For technical reasons, it is difficult to extract bacteria from tumor samples for further experiments, and intestinal flora may not be typical in representing the characteristic of tumor-resident microbiota. To select appropriate bacteria for further research, we analyzed the contents of common subtypes of highly enriched microorganisms in tumors by 2bRAD-M technology (Supplementary Fig. 2A), and selected a total of 9 different subtypes of different bacteria for in vivo pre-experiments. According to the tumor formation and infection of the mice, we finally decided to use *E.coli*: O6(ATCC 25922) for follow-up studies because the incidence of CRLM in model mice using this subtype of bacteria is relatively high, and the severe infection rate (≥25% weight loss) of the mice is lower (Supplementary Table S2).

To confirm the effect of *E.coli* to CRLM, a bioluminescent CRLM model was created by intrasplenic injection of tumor cells derived from a murine primary colon carcinoma (MC38-luc) [16] into a C57BL/6 syngeneic immunocompetent host (6–8 weeks old). One week after the establishment of CRLM models, the mice were orally gavaged with *E.coli* (1 × 10^7^ colony-forming units [CFUs]) every other day for 1 week. We also administered a broad-spectrum antibiotic mix (Abx) to inhibit microbiota growth to create a bacteria-free group. One week after processing, we harvested the liver and examined the tumor microbiota using 16 S rDNA. The *E.coli*-treated mice had a higher abundance of bacteria in the liver than the Ctrl-treated mice, while the Abx-treated mice had significantly reduced microbiota inside the liver. We detected 3.4 × 10^5^ equivalent bacteria per gram of tissue in *E.coli* group samples, whereas only 1.4 × 10^5^ and 6.8 × 10^4^ equivalent bacteria per gram of tissue were detected in Ctrl group and Abx group samples (Supplementary Fig. 2B). Furthermore, whole-body bioluminescence images show that broad-spectrum antibiotic mix reduced the burden of liver metastasis, the detectable tumor numbers of the liver in the *E.coli* group were significantly more than that in the Ctrl group, whereas the Abx group had significantly less detectable tumor numbers of the liver (Fig. 1C and Supplementary Fig. 2B, C). HE staining demonstrated that the tumor cells in the *E.coli* group were more heterogeneous than those in the Ctrl group and had disordered cell morphology and deep nuclear staining (Supplementary Fig. 2D). Macrophages are the most numerous non-parenchymal cells in the liver and an important component of tumor immunity. Further, macrophages can influence the proliferation and metastasis of tumor cells through polarization [17]. Therefore, we assessed the polarization of macrophages within liver metastasis by immunofluorescence staining. The level of M2 macrophage marker CD206 was significantly increased and that of the M1 marker iNOS decreased in the *E.coli* group tumors compared with those in the Ctrl group tumors, whereas that of CD206 was decreased and iNOS increased in the Abx group tumors (Fig. 1D and Supplementary Fig. 2E), gene expression levels of pro-inflammatory cytokines *Tnfa*, *Il6*, *Il1b*, and *Cxcl10* were markedly promoted in the liver tissues from Abx mice compared to Ctrl and *E.coli* mice (Supplementary Fig. 2F), indicating that tumor microbiota could promote the M2 polarization of macrophages within liver metastasis.

To monitor the behavior of the *E.coli* in the tumor visually, we use a gene-edited *E.coli* that carries spontaneous mCherry fluorescence. By orally gavaged mCherry-*E.coli*, we detected the red microbiota in the liver whereas more *E.coli* can be detected in the tumor comparing with paratumor (Fig. 1E). Moreover, higher dose intake (3 × 10^7^ CFU every other day for 1 week) of *E.coli* showed more CD206^+^ M2 macrophages accumulation, which presented a dose-dependent effect in inducing M2 polarization during liver metastasis. While although *E.coli* improved the Treg percentage, no difference was observed between the low or high-dose *E.coli* group (Fig. 1F).

As oral *E.coli* intake could also modulate the gut immune status, which may indirectly affect the progression of liver tumor, we injected *E.coli* through the tail vein instead of oral gavage for providing an *E.coli*_(iv)_ CRLM group. As expected, CD206 and iNOS expression and gene expression levels of pro-inflammatory cytokines in *E.coli*_(iv)_ group were similar to that in mice receiving oral *E.coli* treatment (Fig. 1G, H and Supplementary Fig. 2G).

Together, these results indicate that tumor-resident microbiota facilitates tumor growth by regulating the immune homeostasis of the TME.

### Microbiota promote CRLM by increasing tumor glycolysis and M2 polarization of macrophages

**Fig. 2:**
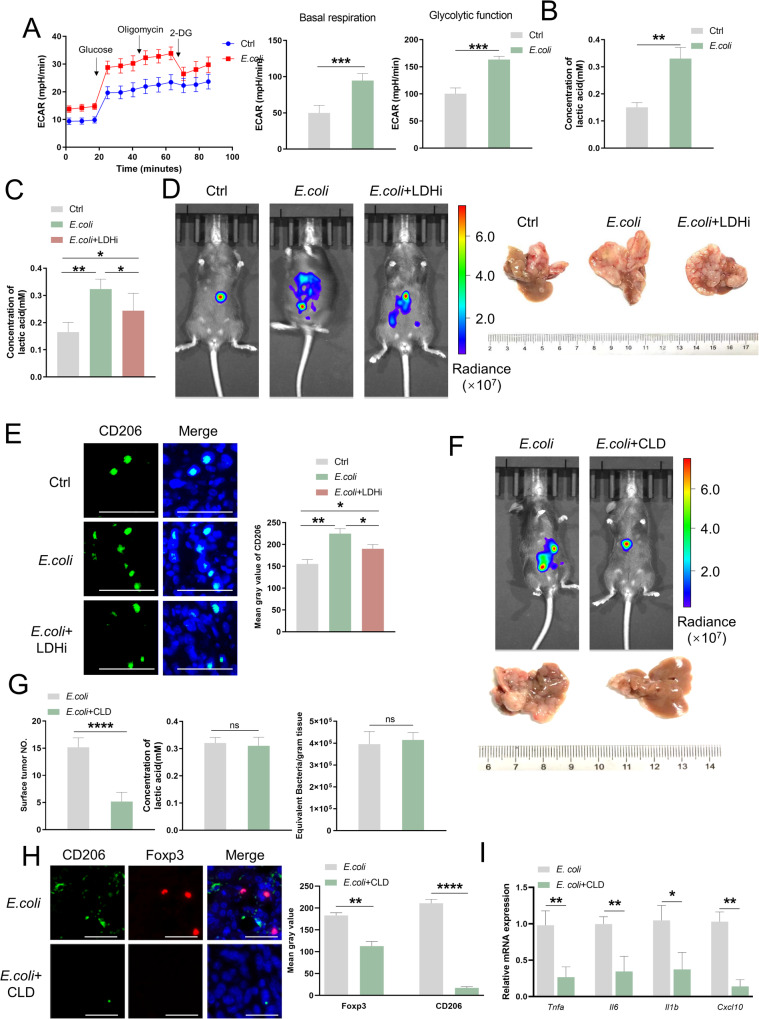
Microbiota promote CRLM by increasing tumor glycolysis and M2 polarization of macrophages. **A** ECAR (mpH/min) of control and *E.coli*-treated MC38 cells. Curves(left) show a change in lactic acid production within 90 min. *n* = 3/group. Each symbol represents the average ECAR. Histogram(mid) represents the basal respiration of control and *E.coli*-treated MC38 cells. Histogram(right) represents glycolytic function control and *E.coli*-treated MC38 cells. Error bars indicate SEM. Statistics were determined using a t-test with significance indicated (***p < 0.001). Data are representative of 3 independent experiments. **B** Lactic acid concentration in culture environment of MC38 cells (*n* = 3) in (**A**). Error bars indicate SEM. Statistics were determined using a t-test with significance indicated (**p < 0.01). **C** Lactic acid concentration in liver metastasis from the control group and mice treated with *E.coli* alone or + LDHi (*n* = 10). Error bars indicate SEM. Statistics were determined using a *t*-test with significance indicated (*p < 0.05, **p < 0.01). **D** Representative whole-body bioluminescence images (left) of mice orthotopically xenografted after intravenous injection with MC38-luc+ cells and representative images of liver metastasis (right) from the control group and mice treated with *E.coli* alone or + LDHi (*n* = 10). Scale bars: 1 cm. **E** Representative co-immunofluorescence images of staining for CD206(M2 marker) and DAPI(nuclear counterstain) in tumors from mice in (**C**). Scale bars, 100 μm. Objective, 10x. **F** Representative whole-body bioluminescence images (up) of mice orthotopically xenografted after intravenous injection with MC38-luc+ cells and representative images of liver metastasis (down) from *E.coli*-injected and *E.coli*/CLD-injected group mice (*n* = 10). Scale bars: 1 cm. **G** The detectable tumor numbers(left), lactic acid concentration(mid), and numbers of microbiota(right) in liver metastasis (*n* = 10) from mice treated with *E.coli* alone or + the CLD liposomes. Error bars indicate SEM. Statistics were determined using a *t*-test with significance indicated (ns, not significant, ****p < 0.0001). **H** Representative co-immunofluorescence images and mean gray value of staining for CD206(M2 marker), Foxp3(Treg marker), and DAPI (nuclear counterstain) in tumors from mice in (**F**). Scale bars, 100 μm. Objective, 10x. **I** Gene expression of *Tnfa, Il6, Il1b*, and *Cxcl10* in liver tissues from mice in (K) (*p < 0.05).

To investigate how tumor-resident microbiota influence tumor cells, we co-cultured *E.coli* (5 × 10^5^ CFUs) in vitro with 2 × 10^6^ MC38 cells for 6 h. The proliferation rate of co-cultured cells was equal to that of control cells by Cell Counting Kit-8(CCK8) assay (*P* = 0.7953) (Supplementary Fig. 3A). Western blot analysis revealed no difference in E-Cadherin or N-Cadherin expression, molecular markers of epithelial-mesenchymal transition (EMT) between *E.coli*-treated and control cells (Supplementary Fig. 3B), transwell migration, and scratch wounding assays showed no difference between Ctrl and *E.coli* group cells (Figure Supplementary Fig. 3C), indicating that microbiota did not affect the proliferation or migration of tumor cells. However, the extracellular acidification rates in both basal respiration and glycolytic conditions were significantly higher in *E.coli-*treated cells than in Ctrl cells (Fig. 2A). Similar results were observed in a lactate concentration assay (Fig. 2B).

The facultative anaerobe *E.coli* was cultured alone in vitro to determine whether its production was responsible for the observed heightened lactate concentration in the co-culture system with MC38. The findings revealed that although E. coli did produce lactate, the amount generated was minimal, with less than 0.1 mmol/L produced within 96 h, far below the *E.coli* and MC38 co-culture system (Supplementary Fig. 3D). Furthermore, *E.coli* oral intake may not upregulated the lactic acid concentration in control mice (no tumor administration). On the other hand, the concentration of lactate in the liver tumors of CRLM model mice was significantly higher compared to its adjacent tissues and the liver tissues of control mice (Supplementary Fig. 3E). This suggests that the predominant source of lactate in vivo was mainly derived from MC38 cells but not *E.coli* itself.

Lactate dehydrogenase inhibitors (LDHi) prevent the conversion of pyruvate to lactate by inhibiting lactate dehydrogenase, a key enzyme in the rate-limiting reaction during glycolysis. Therefore, we conducted in vivo experiments using LDHi (GSK2837808A, MCE) to further validate the role of lactate production in *E.coli-*aggravated CRLM. As expected, LDHi had no effect on the abundance of microbiota in the tumor (Supplementary Fig. 3F) but did reduce the intra-tumoral lactate concentration upregulated by *E.coli* alone (Fig. 2C) and the burden of liver metastasis by noninvasive bioluminescence imaging (Fig. 2D and Supplementary Fig. 3G). Histological examination also indicated a reversal of the histological changes induced by *E.coli* after LDHi treatment (Supplementary Fig. 3H). The gene expression of pro-inflammatory cytokines was markedly inhibited in the liver tissues of *E.coli* mice compared to Ctrl mice. However, these observed results were reversed with the addition of LDHi (Supplementary Fig. 3I).

Since tumor-resident microbiota promotes M2 polarization within liver metastasis, we further examined whether microbiota can regulate CRLM progression via macrophages. We inoculated mice intraperitoneally with clodronate-filled liposomes (CLD) to deplete macrophages [18]. The burden of the liver metastasis in mice treated with *E.coli* and CLD were significantly lower than that in mice treated with *E.coli* alone (Fig. 2F, G), whereas no significant difference in lactate levels and the abundance of microbiota was observed between the two groups (Fig. 2G). Immunofluorescence staining indicated that the M2 macrophage and regulatory T cell (Treg) markers CD206 and Foxp3, respectively, were significantly reduced in the liver metastasis of the *E.coli* and CLD group compared to those of the *E.coli* alone group (Fig. 2H). The gene expression of pro-inflammatory cytokines was markedly decreased in the liver tissues of *E.coli* + CLD mice compared to *E.coli* mice (Fig. 2I).

Tumor-resident Tregs are also important negative regulators of tumor immunity that suppress antitumor T cell activity and assist tumor escape [19]. Therefore, we examined the effect of Tregs by establishing a CRLM model in Foxp3-DTR mice, in which Tregs were depleted upon administration of diphtheria toxin (DT). There were severer burden of liver metastasis and slightly higher detectable tumor numbers, intra-tumoral lactate concentration, the abundance of bacteria, or CD206 expression in Foxp3-DTR mice treated with *E.coli* compared with those of *E.coli*-treated wild-type mice, but without a statistical difference (Supplementary Fig. 4A–C), and no difference of gene expression levels of pro-inflammatory cytokines between these two group mice (Supplementary Fig. 4D). These indicate macrophage M2 polarization, rather than Tregs, is the main downstream effect of *E.coli* and lactate production in CRLM.

### Lactate promotes M2 polarization of macrophages through lactylation of RIG-I^K852^

In order to further understand the role of microbiota in regulating macrophage polarization, we firstly co-cultured *E.coli* (5 × 10^5^ CFUs) in vitro with 2 × 10^6^ Kupffer cells for 6 h. The qPCR showed that there was no significant difference in CD206 and iNOS expression in Kupffer cells+*E.coli* compared with Kupffer cells alone (Supplementary Fig. 5A), there was also no significant difference in the extracellular acidification rates in both basal respiration and glycolytic conditions of Kupffer cells with or without co-culture with *E.coli* (Supplementary Fig. 5B), and the same results were obtained using the same number of bacterial fragments co-culture with MC38 cells Kupffer (Supplementary Fig. 5C, D), indicating that *E.coli* may contribute to macrophage M2 polarization through indirect means.

**Fig. 3:**
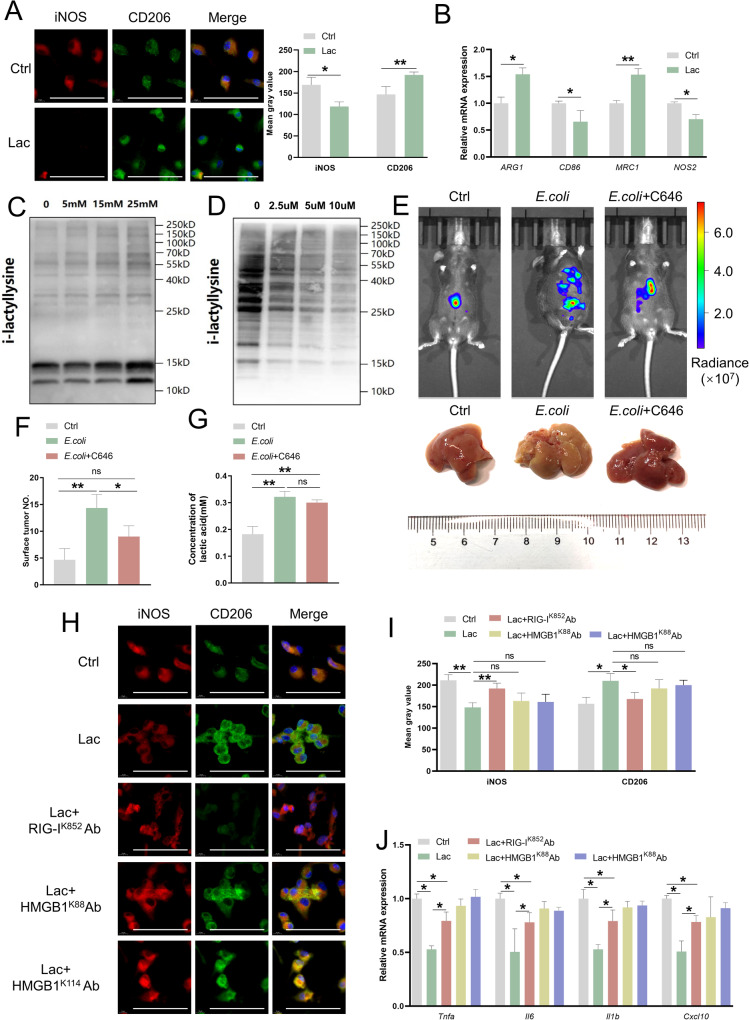
Lactate promotes M2 polarization of macrophages through lactylation of RIG-I^K852^. **A** Representative co-immunofluorescence images and mean gray value of staining for iNOS, CD206, and DAPI in Kupffer cells isolated from C57BL/6 mice after treatment with lactate (5 mmol/l) at day 3. Scale bars, 100 μm. Objective, 10x. **B** Representative histograms show different mRNA levels of *ARG1*, *CD86*, *MRC1* and *NOS2* in Kupffer cells treated with lactate (5 mmol/l). Error bars indicate SEM. Statistics were determined using a t-test with significance indicated (*p < 0.05, ***p < 0.001). Data are representative of 3 independent experiments. **C** Protein lactylation modification in Kupffer cells after treatment with PBS or lactate with different concentrations (5, 15, or 25 mmol/L) at day 3. **D** Protein lactylation modification in liver metastasis from the mice after treatment with PBS or C646 with different concentrations (2.5, 5 or 10 umol/L) at day 3. **E** Representative whole-body bioluminescence images (up) of mice orthotopically xenografted after intravenous injection with MC38-luc+ cells and representative images of liver metastases (down) from control, *E.coli* (1 × 10^9^ CFUs)-treated and *E.coli* (1 × 10^9^ CFUs)-treated + C646 (5 μmol/l)-injected group mice at day 21. *n* = 10/group. Scale bars**:** 1 cm. **F** Histograms of detectable surface tumor numbers in liver metastases from control, *E.coli* (1 × 10^9^ CFUs)-treated and *E.coli* (1 × 10^9^ CFUs)-treated + C646 (5 μmol/l)-injected group mice. *n* = 10/group. Error bars indicate SEM. Statistics were determined using a *t*-test with significance indicated (ns, not significant, *p < 0.05, **p < 0.01). Data are representative of 3 independent experiments. **G** Representative histograms of lactic acid concentration in liver metastasis from the three groups in (**E**). Error bars indicate SEM. Statistics were determined using a *t*-test with significance indicated (ns, not significant; **p < 0.01). Data are representative of 3 independent experiments. **H** Representative co-immunofluorescence images for iNOS, CD206, and DAPI in Kupffer cells treated with lactate (5 mmol/l) + RIG-I^K852^ Ab (1 mmol/l), HMGB1 ^K88^ Ab (1 mmol/l) and HMGB1 ^K114^ Ab (1 mmol/l) or lactate (5 mmol/l) alone at day 3. S Scale bars, 100 μm. Objective, 10x. **I** Mean gray value of staining of (**H**). **J** Gene expression of *Tnfa, Il6, Il1b*, and *Cxcl10* from cells in (**H**) (*p < 0.05).

Then, we treated Kupffer cells or bone marrow-derived macrophages (BMDMs) from B6 mice in vitro with 15 mM lactate. Since the addition of lactic acid can change the pH of the medium, we kept the pH of the medium stable before and after the addition of lactic acid by using Tris buffer. Interestingly, immunofluorescence staining showed that CD206 expression was increased whereas iNOS expression was decreased in lactate-treated macrophages derived from both Kupffer cells and BMDMs (Fig. 3A and Supplementary Fig. 5E). qPCR showed significantly higher levels of M2 markers (*ARG1* and *MRC1*) and lower levels of M1 markers (*CD86* and *NOS2*) in cells treated with lactate (Fig. 3B), the gene expression of pro-inflammatory cytokines was markedly inhibited in cells treated with lactate (Supplementary Fig. 5F, G), suggesting that lactate directly promotes macrophage M2 polarization. We also assessed protein lactylation in lactate-treated macrophages by western blotting using a pan-lactyllysine antibody (Ab). The lactylation level in macrophages increased proportionally to the concentration of lactate (Fig. 3C and Supplementary Fig. 5H).

Lactate, an important epigenetic regulatory molecule, causes lactylation of histones in the presence of the histone acetyltransferase p300 [20]. In order to further clarify whether the effect of microbiota on liver metastasis is caused by lactylation, we intraperitoneally injected C646, an inhibitor of p300, into our *E.coli*-treated mice with CRLM. Western blot showed that the pan-lactylation levels in macrophages in liver metastasis gradually decreased with increasing doses of C646 (Fig. 3D and Supplementary Fig. 5I), indicating that C646 could effectively inhibit protein lactylation in macrophages. Compared with the *E.coli*-treated mice, the detectable tumor numbers and the burden of the liver metastasis by noninvasive bioluminescence imaging in the C646/*E.coli*-treated mice was effectively reduced (Fig. 3E, F), whereas there was no significant difference in lactate levels between the two groups (Fig. 3G). We also treated mice with the Class I histone deacetylases (HDAC3), the most common “erasers” of lactylation [21], and obtained similar results (Supplementary Fig. 5J–M).

We further tried to define the functional lactylation sites related to lactate-mediated regulation of macrophage polarization. We used the mass spectrum data previously published by PTM Bio [20, 21] and searched the public STRING database for lactylation-related proteins and sites that were closely related to macrophage-associated tumor immunity. Several sites were selected, and we obtained customized lactylation-specific Abs separately. According to the protein sequence, two antigenic peptides were designed and synthesized for animal immunization, purification, and detection. At the same time, a non-modified control peptide was designed and synthesized for purification and detection. Dot plot detection of different an anti-retinoic acid-inducible gene 1 (RIG-I)^K852^ Ab polypeptide masses showed the specificity of the Ab (Supplementary Fig. 5N). We then cultured macrophages with these Abs and lactate for 72 h. Immunofluorescence staining showed that CD206 expression was decreased and iNOS expression was increased and gene expression levels of pro-inflammatory cytokines were markedly upregulated in macrophages treated with RIG-I^K852^ Ab compared with that of lactate-treated macrophages, suggesting that RIG-I^K852^ might be a key lactylation site that regulates lactate-induced macrophage polarization. Although lactate promotes macrophage HMGB1 lactylation through p300/CBP [20], we did not observe a significant difference in CD206 or iNOS staining in cells treated with an anti-HMGB1^K88^ Ab or those treated with an anti-HMGB1^K114^ Ab (Fig. 3H–J), suggesting that RIG-I^K852^ might be a key lactylation site that determines the direction of macrophage polarization controlled by lactate.

### Lactylation of RIG-I^K852^ reduces the aggregation of mitochondrial antiviral signaling protein and NF-κB activation

RIG-I is a key RNA sensor that recognizes pathogen-associated molecular patterns and sequentially activates downstream axes to trigger the innate immune response [22]. Upon sensing dsRNA in the cytoplasm, RIG-I changes its conformation and exposes its N-terminal CARD domain to interact with the CARD domain of the adaptor protein mitochondrial antiviral signaling protein (MAVS), which is localized to the mitochondrial outer membrane via its C-terminal transmembrane domain. Once activated, RIG-I forms a complex with MAVS to further activate downstream cytosolic kinases TBK1 and IKK, which to promote phosphorylation of IRF3 and NF-κB and drive their nuclear translocation, leading to the expression of type I IFN, interferon-stimulated genes (ISGs), and other pro-inflammatory cytokines [23].

**Fig. 4:**
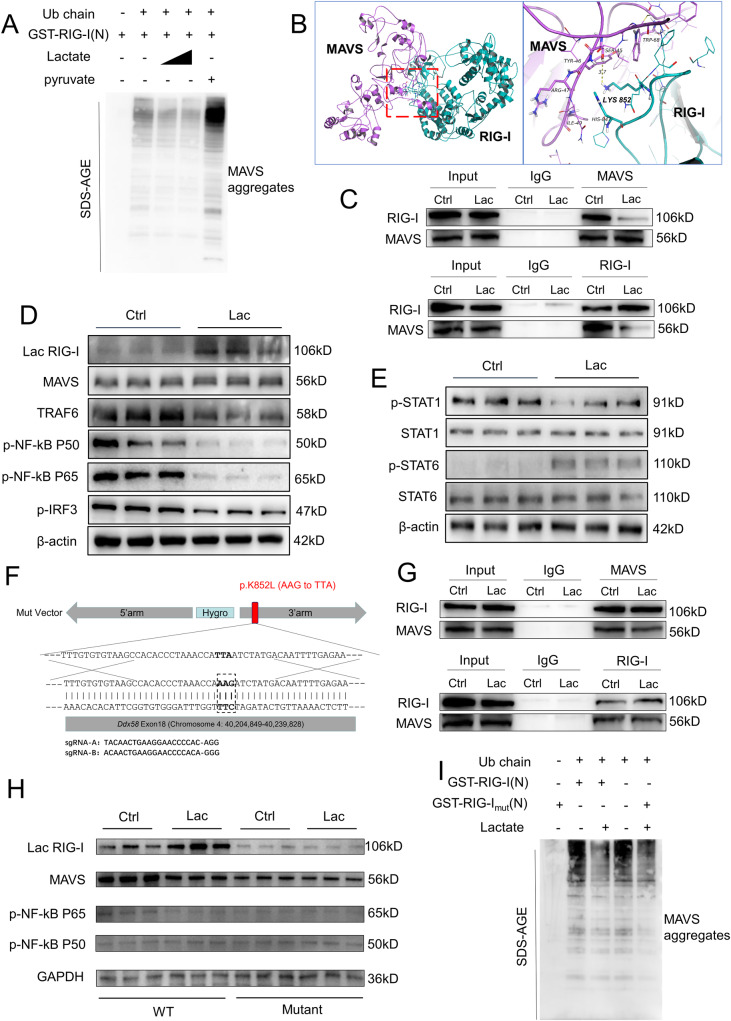
Lactylation of RIG-I^K852^ reduces the aggregation of mitochondrial antiviral signaling protein and NF-κB activation. **A** Immunoblot analysis of in vitro MAVS aggregation. GST-RIG-I(N) was incubated with K63-Ub4 and then with mitochondria isolated from RAW264.7 cells preincubated with or without lactate (5 mM,10 mM) or pyruvate, followed by an analysis of mitochondria extracts using SDD-AGE. **B** Protein-protein docking pose of RIG-I and MAVS before and after Lys852 lactylation predicted by three-dimensional modeling. **C** Co-IP assays confirmed that the binding strength of RIG-I to MAVS was obviously decreased after the lactylation modification. **D** Kupffer cells isolated from C57BL/6 mice were treated with lactate (5 mmol/l) at day 3. Afterward, the protein expression levels of lac-RIG-I, MAVS, TRAF6, p-NF-kB P50, p-NF-kB P65, and p-IRF3 were detected via western blot. **E** The protein expression levels of p-STAT1, STAT1, p-STAT6, and STAT6 in Kupffer cells after treatment with lactate (5 mmol/l) at day 3 were measured by western blot. **F** Mutation of lysine to arginine at site 852 of RIG-I in macrophages by using CRISPR-Cas9 gene editing in RAW264.7 cells. **G** Co-IP assays confirmed that the binding strength of RIG-I to MAVS was no significant difference whether or not treated with lactate after the RIG-I ^K852^ site mutation. **H** Normal RAW264.7 cells or the RAW264.7 cells with RIG-I ^K852^ site mutation were treated with lactate (5 mmol/l) or not at day 3. Afterward, the protein expression levels of lac-RIG-I, MAVS, p-NF-kB P50, and p-NF-kB P65 were detected via western blot. **I** Immunoblot analysis of in vitro MAVS aggregation. GST-RIG-I(N) or GST-RIG-I(N)mu was incubated with K63-Ub4 and then with mitochondria isolated from RAW264.7 or RAW264.7_mut_ cells preincubated with or without lactate (5 mM), followed by an analysis of mitochondria extracts using SDD-AGE. All samples derive from the same experiment or parallel experiments and that gels/blots were processed in parallel.

Purified GST-RIG-I triggered robust MAVS aggregation in the presence of mitochondria and unanchored K63-linked ubiquitin chains in vitro (Fig. 4A), as previously described [24]. Therefore, we examined the potential effect of K852 lactylation on RIG-I structure and function by predicting the structure of RIG-I before and after lactylation using three-dimensional modeling. Lactylation of RIG-I at lysine 852 leads to the loss of the pro-cation-π bond and reduces the aggregation of MAVS (Fig. 4B). RIG-I can also activate other key genes in the ISGylation pathway by activating signal transducer and activator of transcription 1(STAT1) [25]. However, lactylation of RIG-I^K852^ did not affect the interaction between RIG-I and STAT1 because RIG-I and STAT1 interact through hydrogen bonding at RIG-I^549^ and not through polar interaction (Supplementary Fig. 6A).

We further verified the interaction between RIG-I and MAVS by co-immunoprecipitation (CO-IP). Endogenous MAVS was immunoprecipitated from macrophage lysates by a RIG-I antibody, and this interaction was significantly reduced after lactate treatment (Fig. 4C).

We next observed changes in downstream pathways by western blot. The level of lactylated RIG-I was notably increased, whereas the expression of TRAF6, a downstream of RIG-I-MAVS [26], was significantly decreased in lactate-treated macrophages compared with those of control macrophages. Additionally, the phosphorylation levels of NF-κB P50, NF-κB P65, and IRF3, phosphorylated by TRAF6, were also significantly decreased after lactate treatment (Fig. 4D and Supplementary Fig. 6B). As STAT1/STAT6 signaling regulates the polarization of macrophages [27], we observed significantly inhibited STAT1 activation but increased STAT6 activation in macrophages treated with lactate compared with those in control macrophages (Fig. 4E and Supplementary Fig. 6C). To examine the role of p-NF-κB in macrophage polarization, we treated macrophages with the NF-κB agonist TNF-α [28] and lactate. qPCR revealed that *ARG1* and *CD163* expression was downregulated in TNF-α and lactate-treated macrophages compared with that in macrophages treated with lactate alone (Supplementary Fig. 6D), indicating that NF-κB signaling blocks the M2 polarization induced by lactate.

To further examine the effect of RIG-I^K852^ lactylation, we used CRISPR-Cas9 gene editing in RAW264.7 cells to mutate the lysine at RIG-I^K852^ to arginine (Fig. 4F and Supplementary Fig. 6E). PCR result showed that mutations had occurred in the gene-edited cells (Supplementary Fig. S6F). We then treated these mutated RAW264.7 cells with lactate and performed a RIG-I CO-IP. The binding between RIG-I and MAVS was still present after mutation, but the ability of lactate to attenuate this binding was abolished (Fig. 4G). Further, when lysine 852 was mutated, the lactylation of RIG-I was counteracted, and the lactate-mediated decrease of p-NF-κB was reversed (Fig. 4H). Furthermore, we used purified GST-RIG-I from mutated RAW264.7 cells to induce the aggregation of MAVS in vitro. The result indicated no significant difference in MAVS aggregation whether or not treated with lactate in vitro (Fig. 4I and Supplementary Fig. 6G). Therefore, RIG-I^K852^ is the lactylation site that affects macrophage polarization.

### RIG-I depletion reduces inflammasome activation and promotes CRLM progression by decreasing NF-κB phosphorylation and *Nlrp3* transcription

**Fig. 5:**
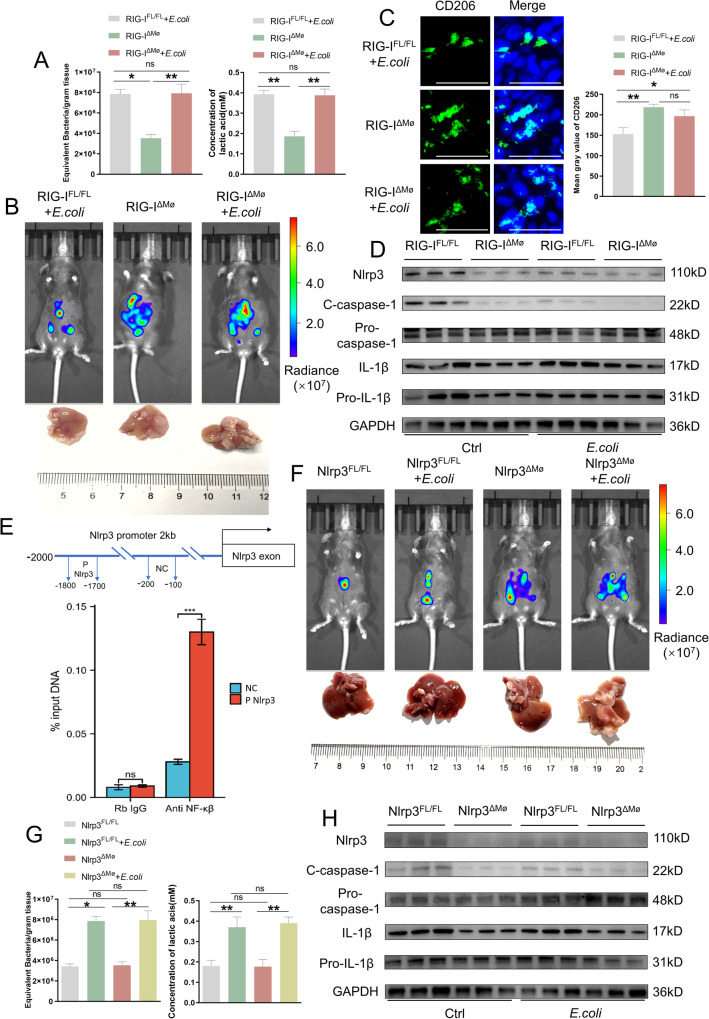
RIG-I depletion reduces inflammasome activation and promotes CRLM progression by decreasing NF-κB phosphorylation and NLRP3 transcription. **A** Representative images of surface tumor numbers in liver metastases from RIG-I^FL/FL^ + *E.coli* (1 × 10^9^CFUs)-treated, RIG-I^ΔMø^, and RIG-I^ΔMø^ + *E.coli* (1 × 10^9^CFUs)-treated mice at day 21. *n* = 10/group. Scale bars: 1 cm. **B** Representative whole-body bioluminescence images (up) of mice orthotopically xenografted after intravenous injection with MC38-luc+ cells and representative images of liver metastasis (down) from the three groups in (**A**). **C** Representative co-immunofluorescence images of staining for CD206 (M2 macrophage marker) and DAPI (nuclear counterstain) in liver metastasis between the three groups in (**A**). Scale bars, 100 μm. Objective, 10x. **D** Kupffer cells were isolated from RIG-I^FL/FL^ + *E.coli* (1 × 10^9^CFUs)-treated, RIG-I^ΔMø^, and RIG-I^ΔMø^ + *E.coli* (1 × 10^9^CFUs)-treated mice, respectively at day 21. Afterward, Nlrp3, C-caspase-1, Pro-C-caspase-1, IL-1β, and Pro-IL-1β expression levels were detected via western blot. **E** Schematic of the CHIP–seq workflow. **F** Representative whole-body bioluminescence images (up) of mice orthotopically xenografted after intravenous injection with MC38-luc+ cells and representative images of liver metastasis (down) from Nlrp3^FL/FL^, Nlrp3^FL/FL^ + *E.coli* (1 × 10^9^CFUs)-injected, Nlrp3^ΔMø^ and Nlrp3ΔMø+*E.coli* (1 × 10^9^CFUs)-injected mice at day 21. *n* = 10/group. Scale bars: 1 cm. **G** Representative histograms of the equivalent bacteria per gram tissue, lactic acid concentration in liver metastasis between the four groups in (**F**). Error bars indicate SEM. Statistics were determined using a *t*-test with significance indicated (ns, not significant, *p < 0.05, **p < 0.01). Data are representative of 3 independent experiments. **H** Kupffer cells were isolated from Nlrp3^FL/FL^, Nlrp3^FL/FL^ + *E.coli* (1 × 10^9^CFUs)-injected, Nlrp3^ΔMø^ and Nlrp3^ΔMø^+*E.coli* (1 × 10^9^CFUs)-injected mice respectively at day 3. Then, the protein expression of Nlrp3, C-caspase-1, Pro-C-caspase-1, IL-1β, and Pro-IL-1β were detected by western blot. All samples derive from the same experiment or parallel experiments and that gels/blots were processed in parallel.

As our results indicated that the lactylation of RIG-I is implicated in macrophage polarization, we assessed the effect of RIG-I expression in macrophages on CRLM progression. We used mice with a macrophage-specific RIG-I knockout (RIG-I^ΔMØ^) to assess the effect of RIG-I expression in macrophages on CRLM progression. The microbiota burden and concentration of lactate were increased in RIG-I^ΔMØ^ or RIG-I^FL/FL^ mice treated with *E.coli* (Fig. 5A); RIG-I knockout significantly promoted the progression of CRLM whether or not treated with *E.coli* (Fig. 5B and Supplementary Fig. S7A). The proportion of M2 macrophages was also increased, and gene expression levels of pro-inflammatory cytokines were markedly inhibited in liver metastasis of RIG-I^ΔMØ^ mice after *E.coli* treatment (Fig. 5C and Supplementary Fig. S7B).

According to previous studies, lactylation of RIG-I results in a decrease in the phosphorylation of the transcription factor NF-κB and translocation of NF-κB into the nucleus, which is the first step in the activation of Nlrp3 inflammatory vesicles in macrophages [29]. The expression of Nlrp3, cleaved caspase-1, and cleaved interleukin (IL)-1β was slightly decreased in macrophages from the tumors of RIG-I^FL/FL^ + *E.coli* group mice and markedly decreased in macrophages from the tumors of RIG-I^ΔMØ^ + *E.coli* and RIG-I^ΔMØ^ Ctrl group mice compared with that in RIG-I^FL/FL^ Ctrl group mice (Fig. 5D and Supplementary Fig. 7C). Kupffer cells isolated from RIG-I^ΔMØ^ mice also showed significantly promoted M2 polarization (Supplementary Fig. 7D), inhibited pro-inflammatory cytokines (Supplementary Fig. S7E), and decreased Nlrp3 signaling pathway activation (Supplementary Fig. 7F) in vitro. We confirmed the binding of NF-κB to the *Nlrp3* promoter by dual luciferase reporter assay (Supplementary Fig. 7G) and ChIP (Fig. 5E). We also verified the binding of NF-κB to the *Nlrp3* promoter using TNF-α, an NF-κB agonist. Western blotting results showed that the binding of NF-κB to the TNF-α was dose-dependent (Supplementary Fig. S7H).

We also constructed macrophage-specific Nlrp3-knockout (Nlrp3^ΔMØ^) mice. Compared with Nlrp3^FL/FL^ Ctrl group mice, CRLM progressed more rapidly in Nlrp3^FL/FL^ + *E.coli* group mice, but slower than Nlrp3^ΔMØ^+*E.coli* and Nlrp3^ΔMØ^ Ctrl group mice and feeding *E.coli* did not accelerate the progression of CRLM in Nlrp3^ΔMØ^ mice (Fig. 5F, G and Supplementary Fig. 7I). The expression of Nlrp3, cleaved caspase-1, and Il-1β was slightly decreased in macrophages from the tumors of Nlrp3^FL/FL^ + *E.coli* mice, and markedly decreased in macrophages from the tumors of Nlrp3^ΔMØ^+*E.coli* and Nlrp3^ΔMØ^ Ctrl mice compared with Nlrp3^FL/FL^ Ctrl groups ((Fig. 5H and Supplementary Fig. 7J). These results reveal that RIG-I in macrophages increases pro-inflammatory cytokine production in macrophages by activating the macrophage Nlrp3 signaling pathway.

### RIG-I^K852^ lactylation in M2 macrophages regulates PD-1^+^ Tregs and CD8^+^ T cells in the TME

**Fig. 6:**
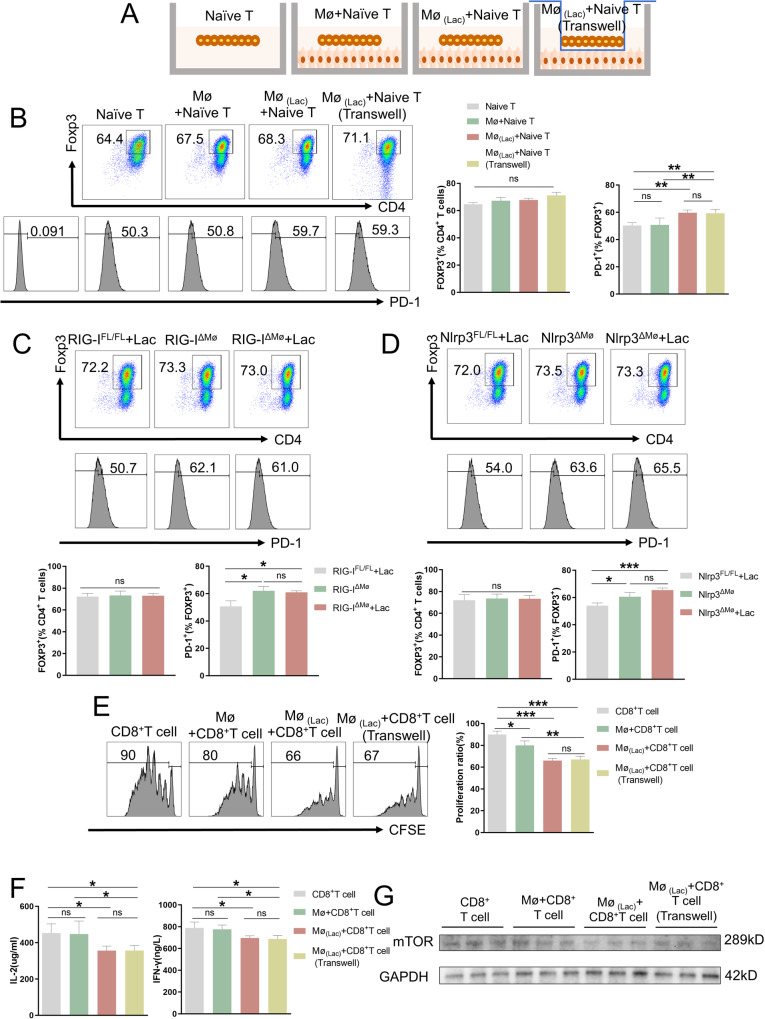
RIG-I ^K852^ lactylation in M2 macrophages regulates PD-1^+^ Tregs and CD8 + T cells in TME. **A** Schematic showing of Naïve T cells did or did not co-cultured with Kupffer cells or lactate-treated Kupffer cells from WT mice with or without using transwell. **B** Representative plots of the percentages of FOXP3^+^ or PD-1^+^ cells(left), a representative histogram of the percentages of FOXP3^+^(mid) or PD-1^+^(right) cells at day 3 of Naïve T cells in (**A**). Error bars indicate SEM. Statistics were determined using a t-test with significance indicated (ns, not significant, **p < 0.01). Data are representative of 3 independent experiments. **C** Representative plots of the percentages of FOXP3^+^(top) or PD^−^1^+^(mid) cells^,^ a representative histogram of the percentages of FOXP3^+^ or PD-1^+^ cells(bottom) at day 3 of Naïve T cells cultured with medium supplemented with supernatant of cultured Kupffer cells from RIG-I^FL/FL^ or RIG-I^ΔMø^ mice with or without lactate. Error bars indicate SEM. Statistics were determined using a t-test with significance indicated (ns, not significant, *p < 0.05). Data are representative of 3 independent experiments. **D** Representative plots of the percentages of FOXP3^+^(top) or PD-1^+^(mid) cells, a representative histogram of the percentages of FOXP3^+^ or PD-1^+^ cells(bottom^)^ at day 3 of Naïve T cells cultured with medium supplemented with supernatant of cultured Kupffer cells from Nlrp3^FL/FL^ or Nlrp3^ΔMø^ mice with or without lactate. Error bars indicate SEM. Statistics were determined using a *t*-test with significance indicated (ns, not significant, *p < 0.05, ***p < 0.001). Data are representative of 3 independent experiments. **E** Representative plots(left) and a representative histogram of the reproductive capacity(right) of CD8^+^ T cells in the presence of PBS or Kupffer cells from WT mice with or without using transwell at day 3. Error bars indicate SEM. Statistics were determined using a t-test with significance indicated (ns, not significant, *p < 0.05, ***p < 0.001). Data are representative of 3 independent experiments. **F** Representative histogram of IL-2(left) and IFN-γ(right) secretion at day 3 of CD8^+^ T cells cultured with medium supplemented with supernatant of cultured Kupffer cells from WT mice with or without lactate. Error bars indicate SEM. Statistics were determined using a t-test with significance indicated (ns, not significant, *p < 0.05). Data are representative of 3 independent experiments. **G** Protein expression levels of mTOR in CD8^+^ T cells cultured with medium supplemented with supernatant of cultured Kupffer cells from WT mice with or without lactate at day 3 were detected by western blot. Data are representative of 3 independent experiments. All samples derive from the same experiment or parallel experiments and that gels/blots were processed in parallel.

To study the effect of lactate-treated macrophages on immune cells in the TME, including Tregs and CD8^+^ T cells, lactate-treated Kupffer cells were co-cultured with naïve T cells from B6 mice using Transwell inserts to eliminate the effect of cell-cell contact (Fig. 6A). Although lactate-treated macrophages did not affect the differentiation of Tregs or the expression of Foxp3, this co-culture with lactate-treated Kupffer cells resulted in higher PD-1 expression in Tregs (Fig. 6B and Supplementary Fig. 8A). We also found that the co-culture promoted the expression of CD39, Ki-67, CD69, CD73, and TGF-β, IL-10, while there was no difference in CTLA-4, GITR, and ICOS (Supplementary Fig. 8B, C). To assess the effect of cytokines secreted by macrophages on the differentiation and function of Tregs, we cultured Tregs with Kupffer cells-conditioned medium. Culturing with media from RIG-I^ΔMØ^ or Nlrp3^ΔMØ^ macrophages did not affect the differentiation of naïve T cells but reduced the expression of PD-1, TGF-β, and IL-10 (Fig. 6C, D, Supplementary Fig. 8D, E).

We also tested the effect of co-culture with lactate-treated macrophages on the activation and expansion of CD8^+^ T cells. CFSE assay showed that lactate-treated macrophages suppressed CD8^+^ Tcell expansion (Fig. 6E) and reduced the expression of antitumor markers, including IL-2 and IFN-γ (Fig. 6F). However, lactate-treated Kupffer cells displayed a stronger suppression ability, and higher of IL-2, IFN-γ, and mTOR expression (Fig. 6G and Supplementary Fig. 8F) were observed. These results reveal that Kupffer cells cultured with lactate can secrete cytokines promoting PD-1 expression in Tregs and reducing the activation of CD8^+^ T cells.

### Natural small-molecule compounds slow the progression of CRLM by inhibiting the lactylation of RIG-I^K852^

**Fig. 7:**
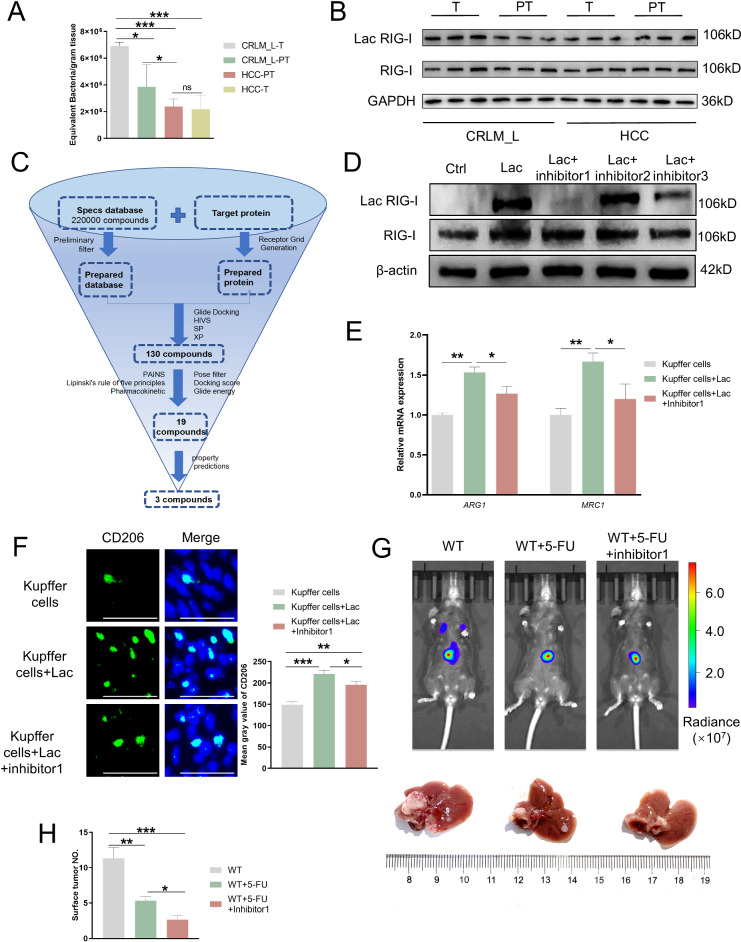
Natural small molecule compounds slow the progression of CRLM by inhibiting the lactytation of RIG-I^K852^. **A** Statistical results of the numbers of microbiota from the tumor in liver metastasis of colorectal cancer (CRLM_L) and its para-tumor (CRLM_L-PT), hepatocellular carcinoma (HCC) and its para-tumor (HCC-PT). *n* = 10/group. Error bars indicate SEM. Statistics were determined using a *t*-test with significance indicated (ns, not significant, *p < 0.05, ***p < 0.001). Data are representative of 3 independent experiments. **B** Protein expression levels of Lac RIG-I and RIG-I in macrophages isolated from CRLM_L, CRLM_L-PT, HCC-T, and HCC-PT respectively were detected via western blot. Data are representative of 3 independent experiments. **C** The discovery and design diagram and binding analysis of three kinds of compounds. **D** Kupffer cells isolated from C57BL/6 mice were cultured with small-molecule compound inhibitor 1 (1 mmol/l), 2 (1 mmol/l), or 3 (1 mmol/l) at day 3. Afterward, Lac RIG-I and RIG-I expression levels were detected via western blot. Data are representative of 3 independent experiments. **E** Representative histograms show different mRNA levels of ARG1 and MRC1 in Kupffer cells treated with lactate (5 mmol/l) alone or combined with inhibitor1 (1 mmol/l) at day 3. Error bars indicate SEM. Statistics were determined using a t-test with significance indicated (*p < 0.05, **p < 0.01, ***p < 0.001). Data are representative of 3 independent experiments. **F** Representative co-immunofluorescence images of staining for CD206 (M2 macrophage marker) and DAPI (nuclear counterstain) in Kupffer cells treated with lactate alone or + inhibitor1 at day 3. Scale bars, 100 μm. Objective, 10x. **G** Representative whole-body bioluminescence images (up) of mice orthotopically xenografted after intravenous injection with MC38-luc+ cells and representative images of liver metastasis from WT, 5-FU (5 mg/kg)-treated alone or 5-FU (5 mg/kg)/inhibitor1 (200 mg/kg)-treated mice. **H** Representative histograms show differences in surface tumor numbers between the three groups in (**G**) at day 21. *n* = 10/group. Scale bars: 1 cm. Error bars indicate SEM. Statistics were determined using a *t*-test with significance indicated (*p < 0.05, **p < 0.01, ***p < 0.001). All samples derive from the same experiment or parallel experiments and that gels/blots were processed in parallel.

We collected clinical samples from patients with CRLM or hepatocellular carcinoma (HCC) and compared them with para-tumor (PT) from CRLM or HCC. We detected 6.9 × 10^6^ equivalent bacteria per gram of tissue in CRLM_L-T samples, 3.9 × 10^6^ equivalent bacteria per gram of tissue were detected in CRLM_L-PT; whereas in HCC, only 2.2 × 10^6^ and 2.1 × 10^6^ equivalent bacteria per gram of tissue was detected in PT or tumor samples (Fig. 7A). It is important to note that the data presented in Fig. 7A is derived from the same set of 20 patient specimens that were monitored in Fig. 1B. CRLM tissues had higher expression of lactylated RIG-I than PT and normal liver tissues, whereas there were significant differences between individual HCC tissues (Fig. 7B). To explore the clinical potential of inhibiting the lactylation of RIG-I combined with the administration of chemotherapeutic drugs, we performed virtual screening of the Specs database based on RIG-I^K852^ and identified approximately 220,000 compounds. We then screened these using high-throughput virtual screening, standard precision screening, and high-precision screening docking methods. Compounds with 10% docking results in all three modes were retained. Overall, 130 compounds were obtained. Nineteen compounds were selected based on the ligand-protein 2D map, docking score, glide energy, number of hydrogen bonds, drug-like parameters, and pharmacokinetic prediction results. Intestinal absorption and water solubility properties were predicted, and 3 compounds that could inhibit the lactylation of RIG-I^K852^ were ultimately identified (Fig. 7C and Supplementary Fig. 9A). Western blot indicated that only inhibitor1 (AJ-64234603004) named as [7-(carboxymethyl)-10-methyl-10H-phenothiazin-2-yl] acetic acid reduced RIG-I lactylation in vitro (Fig. 7D). And their combined state was shown in Supplementary Fig. 9B. The qPCR and immunofluorescence staining showed that the expression of M2 markers (*ARG1* and *MRC1*) and CD206 in lactate-treated Kupffer cells were significantly decreased when treated with inhibitor1; while the levels of M1 markers (*CD86* and *NOS2*) were increased relatively (Fig. 7E, F and Supplementary Fig. 9C), gene expression levels of pro-inflammatory cytokines *Tnfa*, *Il6*, *Il1b*, and *Cxcl10* were markedly upregulated in Kupffer cells treated with inhibitor1 (Supplementary Fig. 9D). The fluorinated analog of uracil, 5-fluorouracil (5-FU), is a fundamental component of chemotherapeutic regimens for the palliative and adjuvant treatment of CRC [30]. Therefore, we used 5-FU alone or combined with inhibitor1 in our CRLM mouse models. The results showed that the burden by noninvasive bioluminescence imaging and detectable tumor numbers of liver metastasis were further reduced when treated with 5-FU/inhibitor1, indicating that the combination treatment delayed the progression of CRLM (Fig. 7G, H).

## Discussion

In this study, we revealed that microbiota accelerated lactate production in CRLM and remarkably changed the immune phenotype. Blocking this pathway may reprogram immunosuppressive cells and improve the antitumor effect of chemotherapy. Our findings highlight the role of tumor-resident microbiota in promoting CRLM and suggest the need for the design and application of novel anti-microbiota-based therapy combined with chemo- or immune therapy in select CRLM patients.

The emergence of immunotherapy has revolutionized cancer treatment, and it is increasingly recognized that the tumor microenvironment, including microbiota, plays a critical role in the response to immunotherapy. The findings of our study suggest that targeting microbiota and its effects on tumor metabolism and immune cells may enhance the efficacy of immunotherapy in CRLM patients.

Microbiota has emerged as a contributor to oncogenesis [31, 32]. Tumor-resident microbiota leads to gut vascular barrier impairment [11] and promotes the survival of circulating tumor cells [14], which are vital steps in metastasis [33]. However, it is unclear whether these microbes are drivers of tumor growth inside the liver or TME is unclear. Using engineered mCherry bacteria, we found that bacteria function within tumors and thus influence the structure of the immune microenvironment. Tumor metabolic reprogramming, widely observed in cancer, increases cancer cell survival and proliferation under stress or energy-limiting conditions [34]. Here, we showed that microbiota can modulate glycolysis and lactate production in tumor cells, suggesting a new role for microbiota as metabolic regulators in supporting cancer cell growth. Tumor metabolism regulates gene expression and immune cell differentiation by histone lactylation [20, 35]. We further identified that microbiota produced lactate, which promoted M2 polarization of macrophages through RIG-I^K852^ lactylation and blocking RIG-I-MAVS-NF-ĸB signaling. Microbiota has been shown to promote colorectal cancer metastasis by stimulating cathepsin K secretion and mediating TLR4-dependent M2 macrophage polarization [36]. The Nlrp3 inflammasome is activated by a broad spectrum of agonists, including both microbiota and sterile triggers [37], and mice with macrophage-specific Nlrp3 knockout had worse CRLM, which might be mediated by IL-18 signaling [38]. We determined that RIG-I increased *Nlrp3* expression by promoting activation of NF-ĸB. We also confirmed that microbiota suppressed CD8^+^ Tcell activation through mTOR and increased the immunosuppressive ability of Tregs by promoting PD-1 expression.

Lactate in the TME affect the function of a variety of immune cells. A previous study showed that lactate inhibit the function of CD8^+^T cells in the TME and promote the function of Tregs, thus leading to tumor progression [39]. Lactate signaling through macrophages may occur through lactylation of histones, which transforms macrophages from the anticancer M1 type to the pro-cancer M2 type [40]. Here, we used customed Abs, conditional KO mice, and CRISPR-Cas9 technology to identify RIG-I^K852^ site as a key lactylation site that determines the direction of macrophage polarization. Surprisingly, unlike other groups, we did not identify lactylation sites on HMGB1 that affected macrophage polarization [41]. This implies that lactate influences progression of different diseases through different sites, and the underlying mechanism of site selection needs further investigation. RIG-I-MAVS signaling triggers an interferon response in tumor cells and the expression of chemokines [42], induces the infiltration of suppressor T cells, and promotes the formation of the TME and tumor growth [43]. A previous study demonstrated that lactate interrupts the RIG-I and MAVS interaction and subsequent MAVS aggregation [24]. Interestingly, we showed how lactate disrupts the RIG-I-MAVS interaction, thereby impairing MAVS aggregation and RLR-mediated signaling. The species and abundance of microbiota were different in different stages of chemotherapy, suggesting that there was also a correlation between microbiota and chemotherapy efficacy [44]. Of note, applying natural small-molecule compounds inhibiting RIG-I^K852^ lactylation suppressed this lactylation and improved the antitumor effect of 5-FU. Thus, our findings suggest that targeting RIG-I lactylation may represent another promising strategy for clinical drug development.

Inflammasomes are cytoplasmic multiprotein complexes that are activated by a variety of pathogen-associated or damage-associated molecular patterns. The function of inflammatory vesicles is to activate caspase-1, which in turn causes maturation and secretion of the pro-inflammatory cytokines IL-1β and IL-18 and induces cellular scorching [45]. We analyzed the Nlrp3 expression after microbiota or lactate treatment and found that the expression of both Nlrp3 and its downstream effectors was reduced in macrophages from RIG-I^ΔMØ^ mice cultured with lactate in vitro. The expression of both Nlrp3 and its downstream effectors was also reduced in macrophages within liver metastasis of RIG-I^ΔMØ^ mice after treatment with *E.coli*, suggesting that lactylation of RIG-I affects Nlrp3 signaling. Activation of Nlrp3 and its downstream effectors is closely related to the occurrence and development of various diseases [46]. Further, severe liver metastasis was observed in mice with Nlrp3-deficient macrophages, which proved that Nlrp3 is involved in regulating tumor-resident microbiota in the TME.

CD8^+^ T cells recognize tumor antigens and have cytotoxic ability toward tumor cells [47]. However, dysfunctional or exhausted CD8^+^ T cells cannot mount an antitumor response [48], which may be related to the regulatory role of macrophages in the TME [49]. Lactate-stimulated macrophages suppressed the activation and proliferation of CD8^+^ T cells, which can cause T cell dysfunction in the TME. Moreover, the Tregs population is highly expanded in the TME, and tumor macrophages can affect Tregs through cytokine secretion [50], tumor region migration [51], and phenotype remodeling [52]. Therefore, we also explored the effect of macrophages on Tregs differentiation and function. Lactate-treated macrophages increased PD-1 expression on Tregs, which was blocked by RIG-I and Nlrp3. Confirming this, Tregs co-cultured with Nlrp3-deficient macrophages had a more potent inhibitory capacity against CD8^+^ T cells, suggesting that the absence of Nlrp3 may be critical in influencing the efficiency of macrophages in promoting Treg differentiation.

This study had few limitations. We did not investigate how microbiota stimulate lactate production in tumor cells, which is a direction that should be further investigated. Additionally, the reasons for the differences in the number and types of microbiotas in the digestive system of residents in different regions are also worthy of further study.

In conclusion, this study suggests that microbiota promote CRLM through the promotion of intra-tumoral glycolysis and formation of lactate. This lactate reduces MAVS and downstream NF-κB activation through RIG-I^K852^ lactylation, which in turn reduces Nlrp3 activation, induces M2 polarization in macrophages, and promotes Treg differentiation. These exert negative immunomodulatory effects to promote tumor progression. The findings of this study provide potential targets for immunotherapy in CRLM patients, including targeting microbiota, lactate production, and the RIG-I-MAVS-NF-κB signaling pathway. As far as we know, this is the first study on the hepatic immune microenvironment of bacteria in tumors, and further research needs to be performed. Taken together, we uncovered a broad range of tumor immunity in the TME, including innate and adaptive immune cells, that are controlled by tumor-resident microbiota and provided insight into the pathophysiological mechanisms of lactylation modifications that regulate macrophage function. Our research provides new ideas and theoretical bases for preventing, diagnosing, and treating related clinical diseases.

## Materials and methods

### Human samples

Human samples were collected from 44 CRC patients: (20 with liver metastasis) and 20 HCC patients who underwent radical resection in the First Affiliated Hospital of Nanjing Medical University. The fresh tissue samples were collected immediately after tumor resection and cryopreserved in liquid nitrogen. Patients had not received any treatment including neoadjuvant radiotherapy, chemotherapy, or traditional Chinese medicine prior to collection, and had no other malignant tumors. The study was approved by the Institutional Ethics Committee of the First Affiliated Hospital of Nanjing Medical University. Informed consent for tissue analysis was obtained before the surgery. The clinical characteristics of patients with CRC are listed in Supplementary Table S1. All the research was performed in accordance with government policies and the Helsinki declaration.

### Mice

Wild-type (WT), FloxP-RIG-I (RIG-I^FL/FL^), FloxP-Nlrp3 (Nlrp3^FL/FL^), Lyz2-Cre RIG-I knockout (RIG-I^ΔMØ^), and Lyz2-Cre Nlrp3 knockout (Nlrp3^ΔMØ^) 6–8-week-old male mice on a C57BL/6 background were used in the experiments. The floxed allele was bred into homozygosity to generate RIG-I^FL/FL^ and Nlrp3^FL/FL^ mice. RIG-I^ΔMØ^ and Nlrp3^ΔMØ^ mice were generated by crossing RIG-I^FL/FL^ or Nlrp3^FL/FL^ mice with Lyz2-Cre mice (all on C57BL/6 background). Littermates with floxed alleles without Cre were used as WT controls (RIG-I^FL/ FL^, Nlrp3^FL/FL^). Foxp3-DTR mice were purchased from the Gempharmatech Co., Ltd.

### Cell lines

Mouse colon cancer cell line MC38(ZQ0933) was purchased from the Shanghai Zhong Qiao Xin Zhou Biotechnology Co. Ltd (Shanghai, China). The *E.coli*: O6 (ATCC 25922) and mCherry-*E.coli* BS00F1-84 strain were purchased from Bio Sci Biotechnology Co. Ltd (Hangzhou, China). Tumor cells were maintained in RPMI 1640 (HyClone, SH30255, GEHealthcare, Chicago, IL) containing 10% (v/v) fetal calf serum (Fcs) (HyClone, SH3007003HI) and 1% (v/v) pen/strep (GIBCO, 15140-122) for 30 generations or more than 3 months before performing experiments. All cell lines in our laboratory are routinely tested for mycoplasma contamination, and the cells used in this study were negative for mycoplasma. None of our cell lines are on the list of commonly misidentified cell lines (International Cell Line Authentication Committee). Human colon tumor and liver tumor tissue samples were obtained from patients who underwent radical colon cancer surgery and partial hepatectomy for simultaneous colon cancer liver metastasis at the First Affiliated Hospital of Nanjing Medical University.

## Supplementary information

Supplementary material

Supplementary Fig. S1

Supplementary Fig. S2

Supplementary Fig. S3

Supplementary Fig. S4

Supplementary Fig. S5

Supplementary Fig. S6

Supplementary Fig. S7

Supplementary Fig. S8

Supplementary Fig. S9

Supplementary Table 1

Supplementary Table 2

## Data Availability

Source data of 16 S rDNA sequencing are provided with this paper. Other data that support the findings are available from the corresponding author upon reasonable request.
