# Correction: Tumor-resident microbiota contributes to colorectal cancer liver metastasis by lactylation and immune modulation

**DOI:** 10.1038/s41388-025-03439-4

**Published:** 2025-06-02

**Authors:** Jian Gu, Xiaozhang Xu, Xiangyu Li, Lei Yue, Xiaowen Zhu, Qiuyang Chen, Ji Gao, Maruyama Takashi, Wenhu Zhao, Bo Zhao, Yue Zhang, Minjie Lin, Jinren Zhou, Yuan Liang, Shipeng Dai, Yufeng Pan, Qing Shao, Yu Li, Yiming Wang, Zibo Xu, Qufei Qian, Tianning Huang, Xiaofeng Qian, Ling Lu

**Affiliations:** 1 Hepatobiliary Center, The First Affiliated Hospital of Nanjing Medical University and Research Unit of Liver Transplantation and Transplant Immunology, Chinese Academy of Medical Sciences, Nanjing, China; 2 Jiangsu Key Laboratory of Cancer Biomarkers, Prevention and Treatment, Collaborative Innovation Center for Cancer Personalized Medicine, Nanjing Medical University, Nanjing, China; 3 Department of General Surgery, The Affiliated BenQ Hospital of Nanjing Medical University, Nanjing, China; 4 Zhejiang Cancer Hospital, Hangzhou Institute of Medicine (HIM), Chinese Academy of Sciences, Hangzhou, China; 5 National Institutes of Health (NIH), New York, NY USA; 6 National and Local Joint Engineering Research Center of Biomedical Functional Materials, School of Chemistry and Materials Science, Nanjing Normal University, Nanjing, China; 7 The Clinical Skills Training Center, The Second Xiangya Hospital of Central South University, Changsha, China; 8 School of Biological Science & Medical Engineering, Southeast University, Nanjing, China; 9 School of Medicine, Southeast University, Nanjing, China

**Keywords:** Cancer immunotherapy, Diagnostic markers

Correction to: *Oncogene* 10.1038/s41388-024-03080-7, published online 18 June 2024

Following the publication of this article, the authors noted that the GAPDH band in Figure 7B was erroneously duplicated from Figure 5H.

The raw data has been reviewed and the corrected Figure 7B is provided below.

Former Fig 7b:
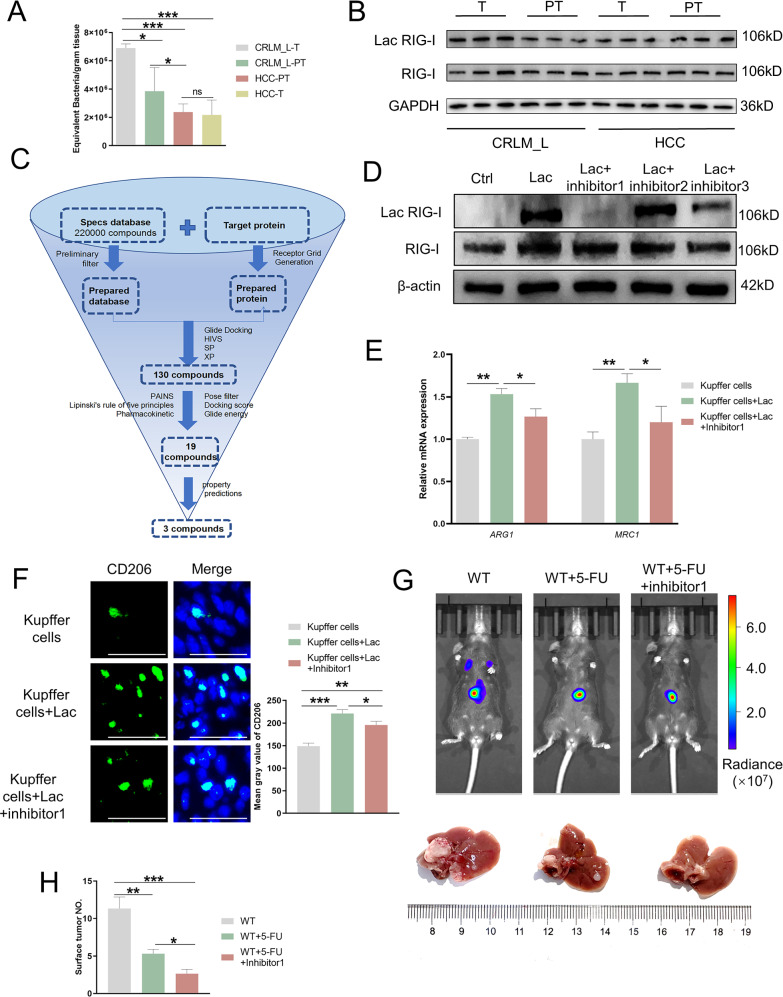


Corrected Fig 7b:
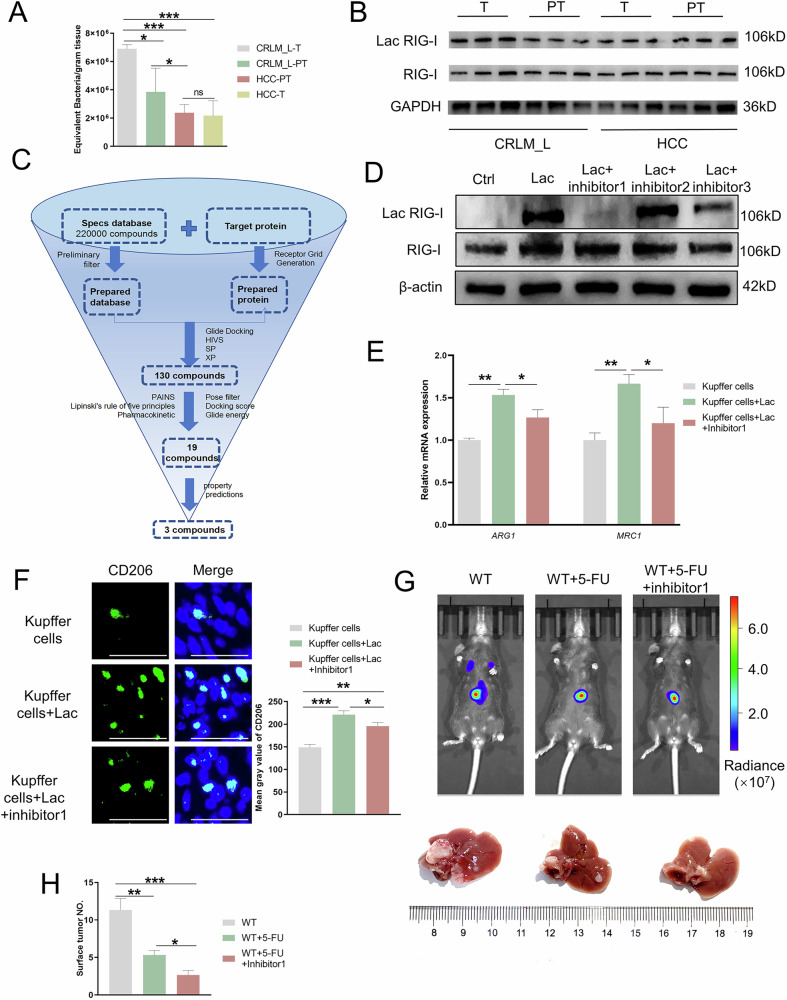


The original article has been corrected.

