# Supplementary material for: Tumor-resident microbiota contributes to colorectal cancer liver metastasis by lactylation and immune modulation
Source: Oncogene. 2024 Jun 18;43(31):2389–404. doi: 10.1038/s41388-024-03080-7 (PMC11281901; doi:10.1038/s41388-024-03080-7)
Supplement: Supplementary file 1 — Supplementary material [file 41388_2024_3080_MOESM1_ESM.docx]

**Tumor-resident microbiota contributes to colorectal cancer liver metastasis by lactylation and immune modulation**

Jian Gu^1,2,5,*^, Xiaozhang Xu^3,5^, Xiangyu Li^3, 5^, Lei Yue^4,5^, Xiaowen Zhu^1,2,5^, Qiuyang Chen^1,2^, Ji Gao^1,2^, Maruyama Takashi^6^, Wenhu Zhao^1,2^, Bo Zhao^7^, Yue Zhang^7^, Minjie Lin^8^, Jinren Zhou^1,2^, Yuan Liang^1,2,9^, Shipeng Dai^1,2^, Yufeng Pan^1,2,10^, Qing Shao^1,2^, Yu Li^1,2^, Yiming Wang^1,2^, Zibo Xu^1,2^, Qufei Qian^1,2^, Tianning Huang^1,2^, Xiaofeng Qian^1,2,*^, and Ling Lu^1,2,3,11,*^

^1^Hepatobiliary Center, The First Affiliated Hospital of Nanjing Medical University and Research Unit of Liver Transplantation and Transplant Immunology, Chinese Academy of Medical Sciences, Nanjing, China

^2^Jiangsu Key Laboratory of Cancer Biomarkers, Prevention and Treatment, Collaborative Innovation Center for Cancer Personalized Medicine, Nanjing Medical University, Nanjing, China

^3^The Affiliated BenQ Hospital of Nanjing Medical University, Nanjing, China

^4^Zhejiang Cancer Hospital, Hangzhou Institute of Medicine (HIM), Chinese Academy of Sciences, Hangzhou, China

^5^These authors contributed equally

^6^National Institutes of Health (NIH), United States

^7^National and Local Joint Engineering Research Center of Biomedical Functional Materials, School of Chemistry and Materials Science, Nanjing Normal University, Nanjing, China

^8^The Clinical Skills Training Center , The Second Xiangya Hospital of Central South University, Changsha, China

^9^School of Biological Science & Medical Engineering, Southeast University, Nanjing, China

^10^School of Medicine, Southeast University, Nanjing, China

^11^Lead contact

^*^Correspondence authors

Correspondence author details:

Jian Gu, The First Affiliated Hospital of Nanjing Medical University, 300 Guangzhou Road, Nanjing 210029, Jiang Su Province, China. Phone: +86 02568303221;

Email: Gujian@njmu.edu.cn

Xiaofeng Qian, The First Affiliated Hospital of Nanjing Medical University, 300 Guangzhou Road, Nanjing 210029, Jiang Su Province, China. Phone: +86 02568303221;

Email: xiaofeng_qian_doc@163.com

Ling Lu, The First Affiliated Hospital of Nanjing Medical University, 300 Guangzhou Road, Nanjing 210029, Jiang Su Province, China. Phone: +86 02568303210;

Email: [lvling@njmu.edu.cn](mailto:lvling@njmu.edu.cn)

**Supplementary Methods**

**Mice model**

Male C57BL/6 mice of 6-8 weeks were anesthetized, and a left upper abdominal transverse incision was made, the abdominal cavity was opened, and the spleen was separated and exposed in the ventral lateral direction. A 1 mL syringe was used to inject approximately 3 mm of tumor cells mixed with *E.coli* suspension from the lower pole of the spleen into the spleen capsule. The concentration of tumor cells in the suspension was 2×10^6^/ml and of *E.coli* was 5×10^5^ CFUs. When the spleen capsule was white and swollen at the injection site, the needle was pulled out, the blood was stopped by compression for 2 minutes, and the abdomen was closed layer by layer. Mice were divided into five groups: Control group (Ctrl); Antibiotic group (Abx): Antibiotic complex (ampicillin 1 g/L, vancomycin 500 mg/L, neomycin 1 g/L, metronidazole 1 g/L) was added to the drinking water of mice and mixed, and drinking was continued until the end of modeling; *E.coli* group (*E.coli*): 100 μL of *E.coli* (1×10^9^ CFUs) was administered orally on day 8–14 of modeling; lactate dehydrogenase inhibitor (LDHi) group: gavage was performed at 6 mg/kg at day 7 and continued until the end of modeling; and clodronate liposome group: 200 μL was intraperitoneally injected one day before modeling and then administered at 3-day intervals until the end of modeling. Lactylation inhibitor C646 group: intraperitoneal injection was performed at 2.5, 5, or 10 μM at day 7 and continued until the end of modeling; and Class I histone deacetylases HDAC3 group: 10, 50, or 100 nM were intraperitoneally injected at day 7 and then until the end of modeling. Liver tissues were collected on Day 21, tumor number were recorded, and the sample size of each experimental group was 5. All the mice were housed under specific ventilated, pathogen-free, and thermostatic conditions with a 12-hour light-dark cycle at 24℃. Food and water were not limited. Then serum and tissues were harvested as described in a previous study^1, 2, 4^. All the animal studies were performed according to the guidelines of the Institutional Animal Use and the Animal Experimentation Ethics Committee of The First Affiliated Hospital of Nanjing Medical University. Natural small molecule compound combined with 5-FU treated mice model: In total 24 C57BL/6 mice bearing tumors were randomly divided into four groups (n=10 per group) and treated as follows: (1) Control group; (2) 5-FU group that received intraperitoneal injection of 5-FU (5 mg/kg) every other day; (3) 5-FU+compound group that received intraperitoneal injection of 5-FU (5 mg/kg) every other day and compound was administered i.p. daily on days 1–10 at 200 mg/kg. Doses and schedules were determined by tolerance studies.

**16S rDNA sequencing**

In total 40-70 mg of tumor tissues were taken, and total DNA was extracted by the Cetyltrimethylammonium Bromide (CTAB) method, and then the quality and concentration of genomic DNA were checked using Qubit.

Absolute quantification-PCR (AQ-PCR) technology uses the standard product with known copy number to make a standard curve, by measuring the cycle threshold (Ct) value of the unknown sample, calculating the starting concentration of the sample from the standard curve, and realizing the absolute quantification of the measured sample gene mRNA, DNA molecular copy number. When assessing the sample, the standard underwent the PCR cycle simultaneously with the unknown sample, and the starting copy number of the unknown sample was obtained according to the Ct value of the unknown sample and combined with the standard curve. Ct refers to the number of amplification cycles passed when the fluorescence signal of the amplification product reaches the set threshold value during PCR amplification, which is reproducible. The fluorescence threshold was taken on the fluorescence signal from the first 15 cycles of the PCR reaction as the fluorescence background signal, and the general fluorescence threshold was defined as 10 times the standard deviation of the fluorescence signal of 3–15 cycles. The standard can be purified plasmid DNA, *in vitro* transcribed RNA, or ssDNA synthesized *in vitro*.

Copy number (X0) calculation method:

Ct=-K logX_0_+b

Note: k represents the standard curve slope, b represents the standard curve intercept=copy number per g sample =X_0_ * 50 uL (eluted volume) / weight assuming the standard curve is: y=-3.481x + 40.123 substitute Ct (18.45) into the linear equation 18.45=-3.481 X + 40.123 X=(18.45-40.123) / (-3.481)=6.226 X_0_=Quantity_Unknown_=10 ^6.226^=1682674

**Kupffer cells extraction, culture, and grouping**

The mice were anesthetized and opened, and the portal vein was separated with T-shaped fixation. There was tension in pulling the lower left adipose tissue moderately to straighten the portal vein. An intravenous indwelling needle (24 g) was inserted 3–4 mm in the portal vein while bending the tip naturally. Then, the needle was slowly withdrawn followed by clamping with hemostatic clips. The perfusion machine was opened, and CMF HBSS was lavaged at 5 mL/min. When the liver was swollen and white, the inferior vena cava was cut, and a large amount of blood was drained out. Lavage was continued with intermittent compression of the inferior vena cava to fill the liver 5–10 times. HBSS containing 0.05% collagenous IV (preheated at 37 °C) was infused at 5 mL/min, and the inferior vena cava was pressed intermittently. The liver was freed and put in a petri dish, and forceps were used to break the liver capsule. The cell suspension was filtered with 70 um grinding filter and centrifuged at 50×g for 3 min at 4°C. The rate of increase was 7, and the rate of decrease was 0. The supernatant was centrifuged at 500×g for 8 min and resuspended in 3 mL 1640 medium to precipitate. Layup: 15 mL centrifuge tube bottom 3 mL of 50% Percoll solution, middle 3 mL of 25% Percoll solution, top 3 mL of cell suspension. After centrifugation at 800×g for 15 min at 4 degrees, the ascending speed was 1, and the descending speed was adjusted to 0 by a brake. Cells in the 2/3 layer were absorbed into a 50mL centrifuge tube (i.e., cells in the 25% and 50% layers), filled with PBS, washed twice, and centrifuged at 500×g for 8 min. The 1640 resuspended precipitate was inoculated into the culture dish, and the non-adherent cells were removed by changing the solution 30 min later. Kupffer cells complete culture medium containing M-CSF was added and spread on a 6-well plate at a density of 1×10^6^/mL. The culture medium was added and placed in an incubator with 5% CO_2_ at 37℃. The treatments were divided into 6 groups: Control group (Ctrl), lactate group (15 mmol/L Lac), RIG-I^K852^Ab group (15 mmol/L Lac+ RIG-I^K852^Ab 3.7 mg/mL), HMGB1^K88^Ab group (15 mmol/L Lac+HMGB1 ^K88^Ab 3.8 mg/mL), HMGB1 ^K114^Ab group (15 mmol/L Lac+HMGB1 ^K114^Ab 1.6 mg/mL), and TNF-α group (15 mmol/L Lac +50 ng/mL TNF-α). The cells were incubated in a 5% CO_2_ incubator at 37℃ for 24 h, and then the proteins or RNA were collected for the next experiment.

**Bone marrow-derived macrophage (BMDM) extraction**

The mice were euthanized, and the femur and tibia were separated. The attached tissue on the bone was removed, and the bone marrow was flushed into a 50 mL centrifuge tube with DMEM complete medium and centrifuged for 5 min at 1250 rpm. Red blood cell lysate was added, mixed, and then centrifuged again.

**Treg differentiation *in vitro***

Mice: We obtained suspensions of murine leukocytes from lymph nodes and spleens. Naive CD4^+^ T cells were then acquired by auto-MACS (Miltenyi, San Diego, CA, USA) based on their CD4^+^/CD62L^+^ surface marker. Then, naive T cells were activated in 96-well plates with completed media supplemented with anti-CD3/28 beads (Dynal beads, 1:1). For the differentiation of p-Tregs, cells were activated in the presence of IL-2 (100 U/mL, R&D Systems) and TGF-β (5 ng/mL, R&D Systems). The completed media is RPMI supplemented with 10% heat-inactivated FBS, 1% antibiotics, 1% HEPES and 0.1% 2-mercaptoethanol. Human: Peripheral blood (PB) leukapheresis products were obtained from volunteers at Nanjing Medical University. Naive human CD4^+^ T cells (CD4^+^CD25^+^) were sort-purified from PB mononuclear cells (PBMCs) (Ficoll-Hypaque, Amersham Biosciences) in a two-step procedure. p-Tregs were stimulated with anti-CD3/CD28 mAb-coated Dynabeads (Life Technologies, Carlsbad, CA) at 1:1 (cell to bead) ratios in the presence of recombinant IL-2 (100 U/mL, R&D Systems). TGF-β (5 ng/mL, R&D Systems) in X-Vivo-15 (BioWhittaker, Walkersville, MD) media supplemented with 10% human AB serum (Valley Biomedical) on day 0. Cells were counted and cultured at the concentration of 0.5×10^6^ cells/mL, and IL-2 (300 U/mL) was renewed every 2 or 3 days. On point days (day 0, 3 or 6), cells were re-suspended at 0.5×10^6^ cells/mL and renewed together with IL-2. Cells were harvested and assayed as listed.

**Co-culture system**

Macrophages M_Ø_ from WT mice, RIG-I^ΔMø^ mice or Nlrp3^ΔMø^ mice were cultured for 48 h using completed media is DMEM supplemented with. supplementation with 10% heat-inactivated FBS, 1% antibiotics, 1% HEPES 0.1% 2-mercaptoethanol, IL-4 (20 ng/mL) and IL-13 (20 ng/mL) to induce differentiation to M2 macrophages. After obtaining a certain purity of M2 macrophages, 5×10^5^/mL of M2 cells and 5×10^5^/mL of naive T cells were co-cultured with or without Transwell, and Treg characteristic activation markers and functional markers were assayed after 3 days of co-culture.

**Bacterial infection of MC38 cells**

MC38 cells were cultured with RPMI complete medium. *E.coli* (5×10^5^ CFUs) were co-cultured with 2×10^6^ MC38 for 6 h in a 5% CO2 incubator at 37℃.

**Virtual screening of Specs database performed based on Lysine No. 852 (K852) on RIG-I protein**

Approximately, 220,000 compound molecules in the Specs database and RIG-I protein in 6KYV were screened by high throughput virtual screening (HTVS), standard precision screening (SP) and high precision screening (XP). The docking results of the three docking modes were 10%, 10%, and 10% respectively. The results showed that 130 compound molecules were obtained from the Specs database, and 19 small molecules were selected based on the ligand-protein 2D map, Docking score, Glide energy, number of hydrogen bonds, drug-like parameters, and pharmacokinetic prediction results. The properties of intestinal absorption and water solubility of these 19 small molecules were predicted, and 3 of them were screened out with high property scores.

**Cell counting kit-8 (CCK8) experiment**

MC38 cells were seeded in 96-well plates, and 100 uL of cell suspension (approximately 5×10^3^ cells) was added to each well. The co-culture model of *E.coli*-infected MC38 cells was established as the experimental group, and MC38 cells without bacterial infection were used as the control group. Five multiple wells were set up in each group, and 10 μL CCK8 detection solution was added to each well, and the cells were incubated for 2 h. The light absorbance value was detected at 450 nm using a microplate reader.

**Extracellular acidification rate (ECAR)**

The above co-cultured cells were prepared into a cell suspension, and 80 μL of cells (approximately 1×10^4^ cells) were seeded into a 96-well Seahorse plate per well. It stood at room temperature for 1 h, and then was put into the incubator for further cultivation for 23 h, 25 uL glucose, oligomycin and 2-deoxyribose were added to probe plate A, B and C, respectively. After correction of probe plate, cell culture plate was added for detection.

**Determination of lactate concentration**

The tumor tissue was prepared into tissue homogenate, and the enzyme working solution and chromogenic agent were prepared according to the instructions. The samples, enzyme working solution and chromogenic agent were added to the plate successively and incubated at 37 °C for 10 minutes, followed by the addition of terminator to terminate the reaction. The reaction solution was 250 μL, and the wavelength of microplate reader was set to 530 nm.

**Histopathology**

Liver tissues were excised and fixed in 10% formalin buffer and subsequently embedded in paraffin. Liver tumor tissue sections were stained with hematoxylin eosin (HE).

**Immunofluorescence and confocal microscopy**

After paraffin sections of liver tumor specimens were dewaxed, antigen repaired, and blocked with serum, the samples were incubated with primary antibodies at 4°C overnight and then incubated with corresponding HRP-labeled secondary antibodies for 50 min at 15–25°C. After washing with PBS three times, Bovine serum albumin was added, and the cells were incubated for 10 min at room temperature in the dark and then washed with Tris buffered saline with Tween-20 three times. 4',6-diamidino-2-phenylindole was dropped for counterstaining, the slices were sealed, and the images were observed and collected under a fluorescence microscope.

**Measurement of lactate**

The tumor tissue was prepared into tissue homogenate, and the supernatant was obtained by centrifugation. The enzyme working solution and chromogenic agent (Nanjing Jiancheng Institute of Biological Engineering) were added to the incubation supernatant. After adding the terminator, the absorbance was measured at the wavelength of 530 nm on the enzyme labeling instrument. The concentration of lactate in the tumor was calculated according to the absorbance.

**CRISPR/Cas9 editing**

A point mutation p.K852L (AAG to TTA) project was introduced into the mouse Ddx58 gene exon 18 of mouse monocyte macrophage leukemia cells RAW 264.7 using CRISPR/Cas9 technology. Single clones were selected after electrotransfection, and the homozygous cells with the point mutation p.K852L (AAG to TTA) in mouse Ddx58 gene exon 18 of mouse macrophage leukemia cells RAW 264.7 were obtained by PCR and sequencing. Single clones were selected after electric conversion, and the homozygous cells with point mutation p.K852L (AAG to TTA) in mouse Ddx58 gene exon 18 of mouse macrophage leukemia cells RAW 264.7 were obtained by PCR and sequencing.

**RIG-I and MAVS: Protein−Protein Docking with RosettaDock**

We used RosettaDock to obtain initial structures for the RIG-I−MAVS complexes. It employs a Monte Carlo (MC) search with low-resolution runs followed by high-resolution refinements. The starting orientations of RIG-I with respect to MAVS were generated by ZDOCK (https://zdock.umassmed.edu/). The docking protocol started with 15 000 steps of a low-resolution rigid-body MC search followed by 500 cycles of refinement. The best binding mode was selected based on Score and visualized with pymol2.2. The lactated Lys852 of RIG-I and MAVS binding mode were generated on wild-type binding mode.

**STAT1 and MAVS: Protein−Protein Docking with RosettaDock**

The method is similar with previous version.

**qPCR**

The treated cells were collected and RNA was extracted. The concentration and purity of RNA were detected by Nanodrop. After reverse transcription into cDNA, Q-PCR system (10 uL) was prepared according to the ratio of pure water: SYBR Mix: pre-primer: post-primer: cDNA= 3.6:5.0:0.2:0.2:1.0, and qPCR was performed. After the reaction, the data were copied for subsequent analysis.

**Western blot analysis**

The treated cells were collected, the radio immunoprecipitation assay lysate was added to extract total protein, and the Bicinchoninic Acid (BCA) method was used for quantification. The same amount of protein was taken for sodium dodecyl sulfate⁃polyacrylamide gel electrophoresis, membrane transformation, and 5% skim milk powder solution blocking for 1 h. The primary antibody (1:1000) was added and incubated at 4℃ overnight. After cleaning with TBST, HRP-labeled secondary antibody (1:2000) was added and incubated at room temperature for 2 h.

**ELISA**

IL-2, IL-10, IFN-γ and TGF-β levels were measured using commercially available enzyme-linked immunosorbent assay (ELISA) kits (BioLegend) according to the manufacturer’s instructions.

**Surface and intracellular staining with flow cytometry**

For intracellular staining of cytokines, cells were stimulated with phorbol myristate acetate (0.25 mg/mL) and ionomycin (0.25 mg/mL) for 5 hours and with brefeldin A (5 mg/mL) for 4 hours. Then, the surface markers, including CD4 and CD25, were stained for 30 min which was followed by further fixation, permeabilization, and stained with Foxp3, IL-10, and TGF-β.

**Co-Immunoprecipitation (CO-IP)**

The cells were collected and washed twice. The cells were lysed with RIPA lysate, and then the protein concentration was determined via BCA. The lysates were clarified with agarose resin for antibody fixation, and the protein content in the filtrate was analyzed after elution of the complex. Western Blotting analysis was performed to determine the expression of binding proteins.

**Database analysis**

The public STRING database collects the proteins and their sites that can undergo lactylation modification and shows the interaction between these proteins. With ‘F4/80’ as the keywords, we screened out some interacting proteins and their sites in the database and then selected three proteins to customize the specific antibodies for lactylation sites.

**Virtual screening**

The Specs database (~204,000 compounds) was used as the screening library. All compounds were first treated with Pipeline Pilot v7.5 (Accelrys) to generate 3D coordination, strip salt, minimize molecule energy, and standardize the chemical table coding. FILTER 2.1.1 from OpenEye was then applied to filter unfavorable compounds using the recommended filter criteria documented in filter_blockbuster.txt provided by OpenEye. Conformations were generated by OMEGA 2.4.6 using the optimized parameters with the FLIPPER module turned on. To explore the clinical potential of inhibiting the lactylation of RIG-I combined with the administration of chemotherapeutic drugs, we performed virtual screening of the Specs database based on RIG-I^K852^ and identified approximately 220,000 compounds. We then screened these using high-throughput virtual screening, standard precision screening, and high-precision screening docking methods. Compounds with 10% docking results in all three modes were retained. Overall 130 compounds were obtained. Nineteen compounds were selected based on the ligand-protein 2D map, docking score, glide energy, number of hydrogen bonds, drug-like parameters, and pharmacokinetic prediction results. Intestinal absorption and water solubility properties were predicted, and three compounds that could inhibit the lactylation of RIG-I^K852^ were identified

**Statistical analyses**

Experimental data were expressed as mean ± standard error (SD). T-test was used for comparison between two groups, and one-way analysis of variance (ANOVA) was used for comparison between multiple groups. *P* < 0.05 was considered statistically significant.

**MAVS aggregation assays**

For *in vitro* MAVS aggregation, crude mitochondria were isolated, and RIG-I activation was detected as previously described^3^. Briefly, each 1 ml of mixture contained 100 ng GST-RIG-I(N) and 50–100 ng ubiquitin chains (K63–Ub4 from Boston Biochem UC-310B) in buffer containing 20 mM HEPES-KOH (pH 7.0) and 10% (v/v) glycerol. After incubation at RT for 10 min in total 10 μL reaction system, 1 μL of reaction mixture was mixed with 10 μg of mitochondrial fraction in 10 μL Buffer B (20 mM HEPES-KOH [pH 7.0], 5 mM MgCl_2_, and 0.25 M D-mannitol) at 30°C for 30 min. The mitochondria fraction was then pelleted at 10,000×g for 10 min and washed twice with Buffer C (20 mM HEPES-KOH at pH 7.4, 0.5 mM EGTA, 0.25 MD-mannitol, and EDTA-free protease inhibitor cocktail) and then subjected semi-denaturing detergent agarose gel electrophoresis.

**Generating RIG-I^arg852^ RAW264.7 cell lines through CRISPR-Cas9 genome engineering**

To clarify that RIG-I^k852^ is the key lactylation modification site that determines the direction of macrophage polarization, we built our editing strategy by using CRISPR-Cas9 system to introduce that mutation in an ATCC-certified RAW264.7 cell line. The overview of our protocol is shown in Figure 4F. First of all, we screened 2 sgRNAs (single guide RNAs) for Mouse Ddx58 (p.K852L) gene and selected sgRNA1(TACAACTGAAGGAACCCCACAGG) and sgRNA2(ACAACTGAAGGAACCCCACAGGG) as the candidate because a Cas9 protein with this sgRNA guide creates a site-specific double-strand break downstream of the genomic site of the target mutation. After the cell test is qualified, the passaging ability is normal, and the genotype test is qualified, the gRNA single chain is synthesized with Oligo, the cells of the formal project group were mixed with the system composed of gRNA, Cas9 protein complex and Oligo, and the cells of the positive control group were transferred into the EGFP plasmid. After 24 hours, the efficiency of the electrotransfection and whether there was any error in the process of electrotransfection were roughly judged according to the fluorescence expression of the positive control group. Twenty-four hours after electrotransfection, the cell poll was sent to detect the efficiency of gRNA and Oligo in the transfection, and the mutation peak was determined according to the sequencing peak map. If the cell pool test result was not satisfactory, the next step was determined after considering whether the power transfer operation, cell, and protocol were abnormal. Then, the cells obtained after monoclonal expansion of positive clones were screened.

**References**

1 Gu J, Zhou J, Chen Q, Xu X, Gao J, Li X *et al*. Tumor metabolite lactate promotes tumorigenesis by modulating MOESIN lactylation and enhancing TGF-beta signaling in regulatory T cells. Cell Rep 2022; 39: 110986.

2 Wang Q, Zhou H, Bu Q, Wei S, Li L, Zhou J *et al*. Role of XBP1 in regulating the progression of non-alcoholic steatohepatitis. J Hepatol 2022; 77: 312-325.

3 Zhang W, Wang G, Xu ZG, Tu H, Hu F, Dai J *et al*. Lactate Is a Natural Suppressor of RLR Signaling by Targeting MAVS. Cell 2019; 178: 176-189.e115.

4 Zhang X, Shen J, Man K, Chu ES, Yau TO, Sung JC *et al*. CXCL10 plays a key role as an inflammatory mediator and a non-invasive biomarker of non-alcoholic steatohepatitis. J Hepatol 2014; 61: 1365-1375.

**Supplementary Figure legends**

**Supplementary Figure S1. Specific microbiota of tumor in CRC_C, CRLM_C, and CRLM_L. related to Figure 1.** (A) The fraction of microbiota in tumor samples from CRC patients with or without liver metastasis by 16s rDNA sequencing. Each color represents a kind of microbiota, and the length of each color represents the abundance of the microbiota in each tumor samples. (B) The distribution of microbiota in tumor samples from CRC-C, CRLM-C, and CRLM-L, respectively, each column represents a kind of microbiota. (C) Overview of the similarity in the composition of microbiota from the tumor in CRC-C (orange, n=24), CRLM-C (red, n=20), and CRLM-L (green, n=20). Every dot represents a kind of microbiota. (D) The specific distribution of microbiota from the tumor between CRC-C and CRLM-C, each dot represents a kind of microbiota. (E) The numbers of microbiota from the tumors (n=20) between CRC-L and CRLM-L, CRC-C, and CRC-PT we collected detected by qPCR. Error bars indicate SEM. Statistics were determined using a t-test with significance indicated (ns, not significant. ***p < 0.001).

**Supplementary Figure S2. CRLM has significant amounts of microbiota, which promotes disease progression related to Figure 1.** (A) The fraction of microbiota in tumor samples from CRC patients with or without liver metastasis by 2bRAD-M. Each color represents a subtype of microbiota, and the length of each color represents the abundance of the microbiota in each tumor samples. (B) The numbers of microbiota from the tumors(n=10) in the tumor from control, antibiotics-treated, and E.coli-treated group mice by qPCR. Error bars indicate SEM. Statistics were determined using a t-test with significance indicated (*p < 0.05, **p < 0.01). (C) The detectable tumor numbers in liver metastasis from the mice(n=10) in (B). Error bars indicate SEM. Statistics were determined using a t-test with significance indicated (*p < 0.05, ***p < 0.001). (D) Representative light microscopy images of H&E-stained of liver metastasis from mice(n=10) in (B). Scale bars, 50 μm. Objective, 5x. (E) Representative co-immunofluorescence images of staining for iNOS(M1 marker) and DAPI(nuclear counterstain) liver metastasis from mice in (B). Scale bars, 100 μm. Objective, 10x. (F) Gene expression of Tnfa, Il6, Il1b, and Cxcl10 in liver tissues from mice in (B) (*p < 0.05). (G) Gene expression of Tnfa, Il6, Il1b, and Cxcl10 in liver tissues from mice in (Figure 1G) (*p < 0.05).

**Supplementary Figure S3. Microbiota promote CRLM by increasing tumor glycolysis. Related to Figure 2.** (A) OD value(450nm) of control and E.coli-treated MC38 cells detected by CCK8 assay. Curves show a change in proliferation ability within 96h. n=3/group. Each symbol represents the average OD value. Error bars indicate SEM. Statistics were determined using a t-test with significance indicated (ns, not significant). Data are representative of 3 independent experiments. (B) The expression levels of E-cadherin and N-cadherin from MC38 cells in (A). Data are representative of 3 independent experiments. (C) Transwell migration(left) and scratch wounding assays(right) of MC38 cells in (A). Scale bars, 200 μm. (D) Lactic acid concentration in culture environment of MC38 cells co-cultured with E.coli, E.coli alone and Ctrl groups within 96h. (E) The concentration of lactate in the liver tumors and paratumor tissues of CRLM model mice and liver tissues of mice gavaged or not(n=3). (F) The numbers of microbiota from the tumors(n=10) in the tumor from control group mice, mice treated with E.coli alone or + LDHi by qPCR. Error bars indicate SEM. Statistics were determined using a t-test with significance indicated (ns, not significant, **p < 0.01). (G) The detectable tumor numbers in liver metastasis(n=10) from control group mice, mice treated with E.coli alone or + LDHi. Error bars indicate SEM. Statistics were determined using a t-test with significance indicated (ns, not significant, *p < 0.05, **p < 0.01). (H) Representative light microscopy images of H&E-stained of liver metastasis(n=10) from control group mice, mice treated with E.coli alone or + LDHi. Scale bars, 50 μm. Objective, 5x. (I) Gene expression of Tnfa, Il6, Il1b, and Cxcl10 in liver tissues from mice in (F) (*p < 0.05). All samples derive from the same experiment or parallel experiments and that gels/blots were processed in parallel.

**Supplementary Figure S4. Microbiota do not promote CRLM by Tregs. Related to Figure 2.** (A) Representative whole-body bioluminescence images (left) of mice orthotopically xenografted after intravenous injection with MC38-luc+ cells and representative images of liver metastasis (right) from E.coli-injected WT mice and E.coli-injected Foxp3-DTR mice(n=10). Scale bars: 1 cm. (B)The detectable tumor numbers(left), lactic acid concentration(mid), and numbers of microbiota(right) in liver metastasis(n=10) from E.coli-treated WT mice and E.coli-treated Foxp3-DTR mice. Error bars indicate SEM. Statistics were determined using a t-test with significance indicated (ns, not significant). (C) Representative co-immunofluorescence images and mean gray value of staining for CD206 (M2 marker), Foxp3 (Treg marker), and DAPI (nuclear counterstain) in tumors from mice in (K). Scale bars, 100 μm. Objective, 10x. (D) Gene expression of Tnfa, Il6, Il1b, and Cxcl10 in liver tissues from mice in (A) (ns, not significant).

**Supplementary Figure S5. Lactate promotes M2 polarization of macrophages through lactylation of RIG-IK852. Related to Figure 3.** (A) Representative histograms show mRNA levels of INOS and CD206 in Kupffer cells whether co-cultured with E-coli (1x109 CFUs). Error bars indicate SEM. Statistics were determined using a t-test with significance indicated (ns, not significant). Data are representative of 3 independent experiments. (B) ECAR (mpH/min) of Kupffer cells and E.coli-treated Kupffer cells. Curves show a change in lactic acid production within 90min. n=3/group. Each symbol represents the average ECAR. Statistics were determined using a t-test with significance indicated (ns, not significant). Data are representative of 3 independent experiments. (C) Representative histograms show mRNA levels of INOS and CD206 in Kupffer cells whether co-cultured with fragment of E-coli (1x109 CFUs). Error bars indicate SEM. Statistics were determined using a t-test with significance indicated (ns, not significant). Data are representative of 3 independent experiments. (D) ECAR (mpH/min) of Kupffer cells and fragment of E.coli-treated Kupffer cells. Curves show a change in lactic acid production within 90min. n=3/group. Each symbol represents the average ECAR. Statistics were determined using a t-test with significance indicated (ns, not significant). Data are representative of 3 independent experiments. (E)Representative co-immunofluorescence images and mean gray value of staining for iNOS (M1 macrophage marker), CD206 (M2 macrophage marker), and DAPI (nuclear counterstain) in bone marrow-derived macrophages (BMDMs) isolated from C57BL/6 mice after treatment with lactate (5mmol/l) at day 3. Scale bars, 100 μm. Objective, 10x. (F) Gene expression of Tnfa, Il6, Il1b, and Cxcl10 from cells in (Figure 3A)(*p < 0.05).(G)Gene expression of Tnfa, Il6, Il1b, and Cxcl10 in liver tissues from mice in (E)(*p < 0.05). (H) The relative indensity of protein expression in (Figure 3C). Error bars indicate SEM. Statistics were determined using a t-test with significance indicated (**p<0.01). Data are representative of 3 independent experiments. (I) The relative indensity of protein expression in (Figure 3D). Error bars indicate SEM. Statistics were determined using a t-test with significance indicated (*p<0.05, **p<0.01, ***p<0.001). Data are representative of 3 independent experiments. (J) Protein lactylation modification in liver metastasis from mice after treatment with PBS or HDAC3 (10, 50, or 100 nmol/L) at day 3. Error bars indicate SEM. Statistics were determined using a t-test with significance indicated (*p<0.05, **p<0.01). Data are representative of 3 independent experiments. (K) Representative whole-body bioluminescence images (up) of mice orthotopically xenografted after intravenous injection with MC38-luc+ cells and representative images of detectable surface tumor numbers in liver metastases (down) from control, E.coli (1x109 CFUs)-treated and E.coli (1x109 CFUs)-treated + HDAC3 (50 nmol/l)-injected group mice at day 21. n=10/group. Scale bars: 1 cm. (L) The histograms of detectable surface tumor numbers in (K). Error bars indicate SEM. Statistics were determined using a t-test with significance indicated (ns, not significant, *p<0.05, **p<0.01). (M) Representative histograms of lactic acid concentration in liver metastasis from the three groups in (K). Error bars indicate SEM. Statistics were determined using a t-test with significance indicated (ns, not significant, **p<0.01). Data are representative of 3 independent experiments. N) Dot plot detection of different K852la Ab polypeptide mass.

**Supplementary Figure S6. Lactylation of RIG-IK852 reduces the aggregation of mitochondrial antiviral signaling protein and NF-κB activation. Related to Figure 4.** (A) Protein-protein docking pose of STAT1 and MAVS before and after Lys852 lactylation predicted by three-dimensional modeling. (B) The mean gray value of protein expression in (Figure 4D) (ns, not significant, **p<0.01, *p<0.05). (C) Mean gray value of protein expression in (Figure 4E) (ns, not significant, **p<0.01, *p<0.05). (D)Representative histograms show different mRNA levels of ARG and CD163 in Kupffer cells treated with lactate (5 mmol/l) alone or combined with TNF-a (1 mmol/l). Error bars indicate SEM. Statistics were determined using a t-test with significance indicated (*p<0.05, **p<0.01, ***p<0.001). Data are representative of 3 independent experiments. (E) A sequence of the final targeting vector, the purple background represents the mutation location. (F) PCR amplicons of the targeted region from 1B7 (edited cells), WT (nonedited cells), and water. (G) Designed Primers targeted to the gRNA of the Mouse Ddx58 gene and its nearby genomic sequence for sequencing. The result of 1B7 sequencing for the location of the mutation indicates the effectiveness of the mutation. (H) The mean gray value of protein expression in (Figure 4H) (ns, not significant, **p<0.01, *p<0.05).

**Supplementary Figure S7. RIG-I depletion reduces inflammasome activation and promotes CRLM progression by decreasing NF-κB phosphorylation and NLRP3 transcription. Related to Figure 5.** (A) Representative images of detectable surface tumor number in liver metastases from mice in (Figure 5A). (B) Gene expression of Tnfa, Il6, Il1b, and Cxcl10 in liver tissues from mice in (Figure 5A) (ns, not significant, *p < 0.05). (C) The mean gray value of protein expression in (Figure 5D) (ns, not significant, **p<0.01, *p<0.05). (D) Representative co-immunofluorescence images and mean gray value of staining for iNOS (M1 macrophage marker), CD206 (M2 macrophage marker), and DAPI (nuclear counterstain) in Kupffer cells isolated from C57BL/6 mice or RIG-IΔMø mice after treatment with lactate at day 3. Scale bars, 100 μm. Objective, 10x. (E) Gene expression of Tnfa, Il6, Il1b, and Cxcl10 in liver tissues from mice in (D)(**p<0.01, *p<0.05). (F) Protein expression levels and mean gray value of Nlrp3, C-caspase-1, Pro-caspase-1, IL-1β, and Pro-IL-1β in Kupffer cells isolated from C57BL/6 mice or RIG-IΔMø mice after treatment with lactate at day 3 were detected by western blot. (ns, not significant, **p<0.01, *p<0.05). (G) Dual luciferase reporter assay of Nlrp3 and NF-κb. (H) Kupffer cells isolated from C57BL/6 mice were cultured with different doses of TNF-a (1ng, 2ng, 3ng, 4ng, 5ng) at day 3. Then the protein expression level of Nlrp3 was detected via western blot. (I) Representative images of detectable surface tumor number in liver metastases from mice in (Figure 5F). (J) The mean gray value of protein expression in (F) (ns, not significant, **p<0.01, *p<0.05). All samples derive from the same experiment or parallel experiments and that gels/blots were processed in parallel.

**Supplementary Figure S8. RIG-I^K852^ lactylation in M2 macrophages regulates PD-1^+^ Tregs and CD8+ T cells in TME. Related to Figure 6.** (A) Representative plots of the percentages of CD4^+^, CD25^+^, FOXP3^+,^ and PD-1^+^ live cells at day 3 of Naïve T cells in Fig. 6A. (B) Representative histogram of the percentages of CD39, Ki-67, CTLA-4, CD69, CD73, GITR or ICOS cells at day 3 of Naïve T cells in Fig. 6A. Error bars indicate SEM. Statistics were determined using a t-test with significance indicated (ns, not significant, *p<0.05, **p<0.01). Data are representative of 3 independent experiments. (C) Representative histogram of IL-10 and TGF-β secretion at day 3 of Naïve T cells in Fig. 6A. Error bars indicate SEM. Statistics were determined using a t-test with significance indicated (ns, not significant, *p<0.05, **p<0.01). Data are representative of 3 independent experiments. (D) Representative histogram of IL-10 and TGF-β secretion at day 3 of Naïve T cells cultured with medium supplemented with supernatant of cultured Kupffer cells from RIG-I^FL/FL^ or RIG-I^ΔMø^ mice with or without lactate. Error bars indicate SEM. Statistics were determined using a t-test with significance indicated (ns, not significant). Data are representative of 3 independent experiments. (E) Representative histogram of IL-10 and TGF-β secretion at day 3 of Naïve T cells cultured with medium supplemented with supernatant of cultured Kupffer cells from Nlrp3^FL/FL^ or Nlrp3^ΔMø^ mice with or without lactate. Error bars indicate SEM. Statistics were determined using a t-test with significance indicated (ns, not significant, *p<0.05). Data are representative of 3 independent experiments. (F) Mean gray value of mTOR of Figure 6G. Error bars indicate SEM. Statistics were determined using a t-test with significance indicated (ns, not significant, *p<0.05, **p<0.01). Data are representative of 3 independent experiments.

**Supplementary Figure S9. Natural small molecule compounds slow the progression of CRLM by inhibiting the lactytation of RIG-I^K852^. Related to Figure 7.** (A) Structure of 3 compounds screened from Fig 7C. (B) Docking pose of inhibitor1 in the allosteric pocket of RIG-I. Surface plot (left): carbon (green), nitrogen (blue), oxygen (red), polar hydrogen (white). 3D interaction plot (right): hydrogen bonds (yellow dashes), pi-pi stacking interaction (blue dashes). (C) Representative histograms show different mRNA levels of CD86 and NOS2 in Kupffer cells treated with lactate (5mmol/l) alone or combined with inhibitor1 (1mmol/l) at day 3. Error bars indicate SEM. Statistics were determined using a t-test with significance indicated (*p<0.05, **p<0.01, ***p<0.001). Data are representative of 3 independent experiments. (D) Gene expression of *Tnfa, Il6, Il1b*, and *Cxcl10* in liver tissues from mice in (Figure 7F) (ns, not significant, *p < 0.05).

**Supplementary Table legends**

**Supplementary Table 1. CRLM has significant amounts of microbiota, which promotes disease progression. Related to Figure 1.**

The information about twenty-four Chinese patients with non-metastatic CRC and twenty Chinese patients with liver-metastatic CRC.

**Supplementary Table 2. The tumor formation rate and serve infection rate of CRLM model in mice were established by different bacteria. Related to Figure 1.**

Each group contained 5 mice.
