# Supplementary figures and images for: Tumor-resident microbiota contributes to colorectal cancer liver metastasis by lactylation and immune modulation
Source: Oncogene. 2024 Jun 18;43(31):2389–404. doi: 10.1038/s41388-024-03080-7 (PMC11281901; doi:10.1038/s41388-024-03080-7)

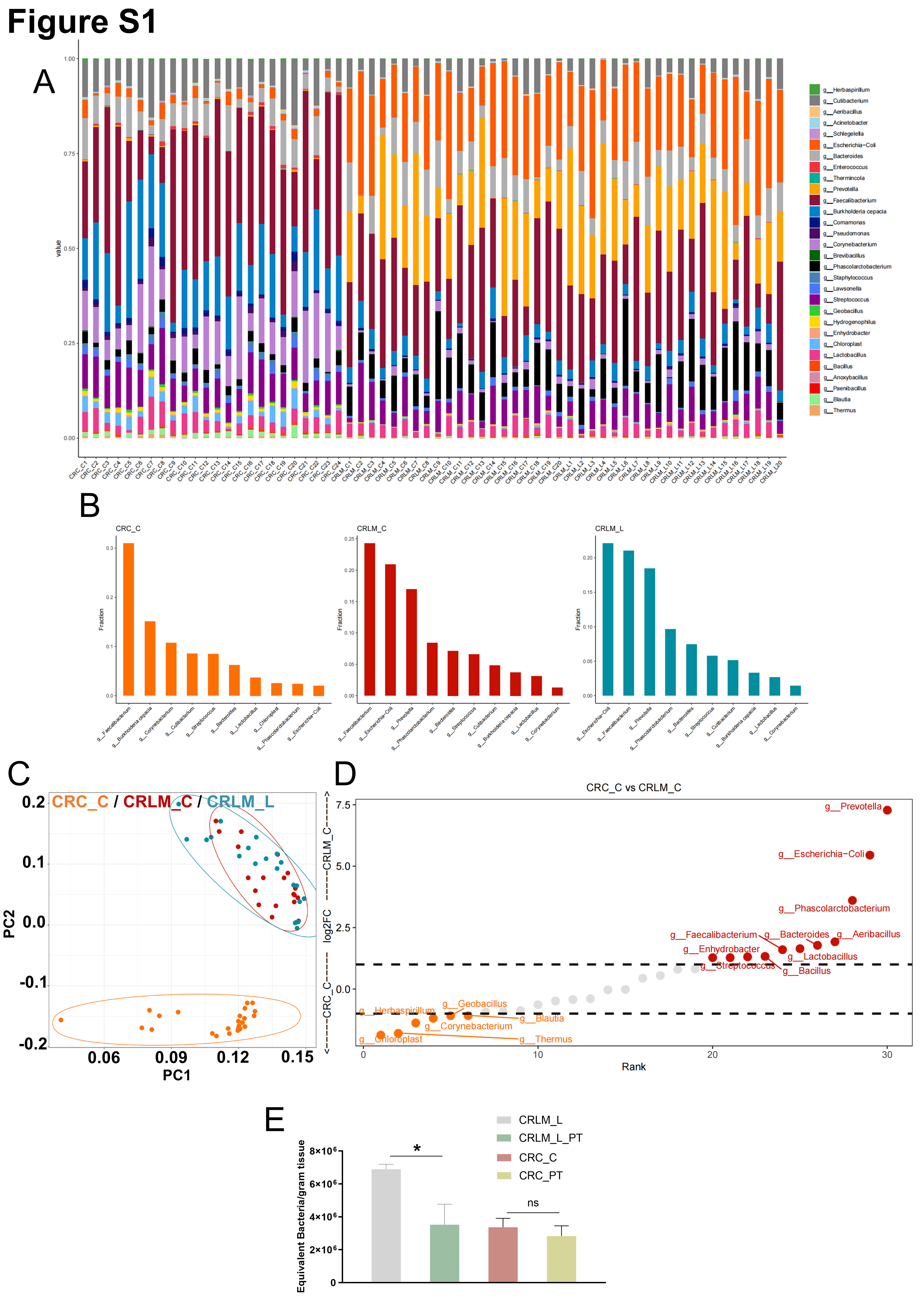

Supplement: Supplementary file 2 — Supplementary Fig. S1 [file 41388_2024_3080_MOESM2_ESM.tif]

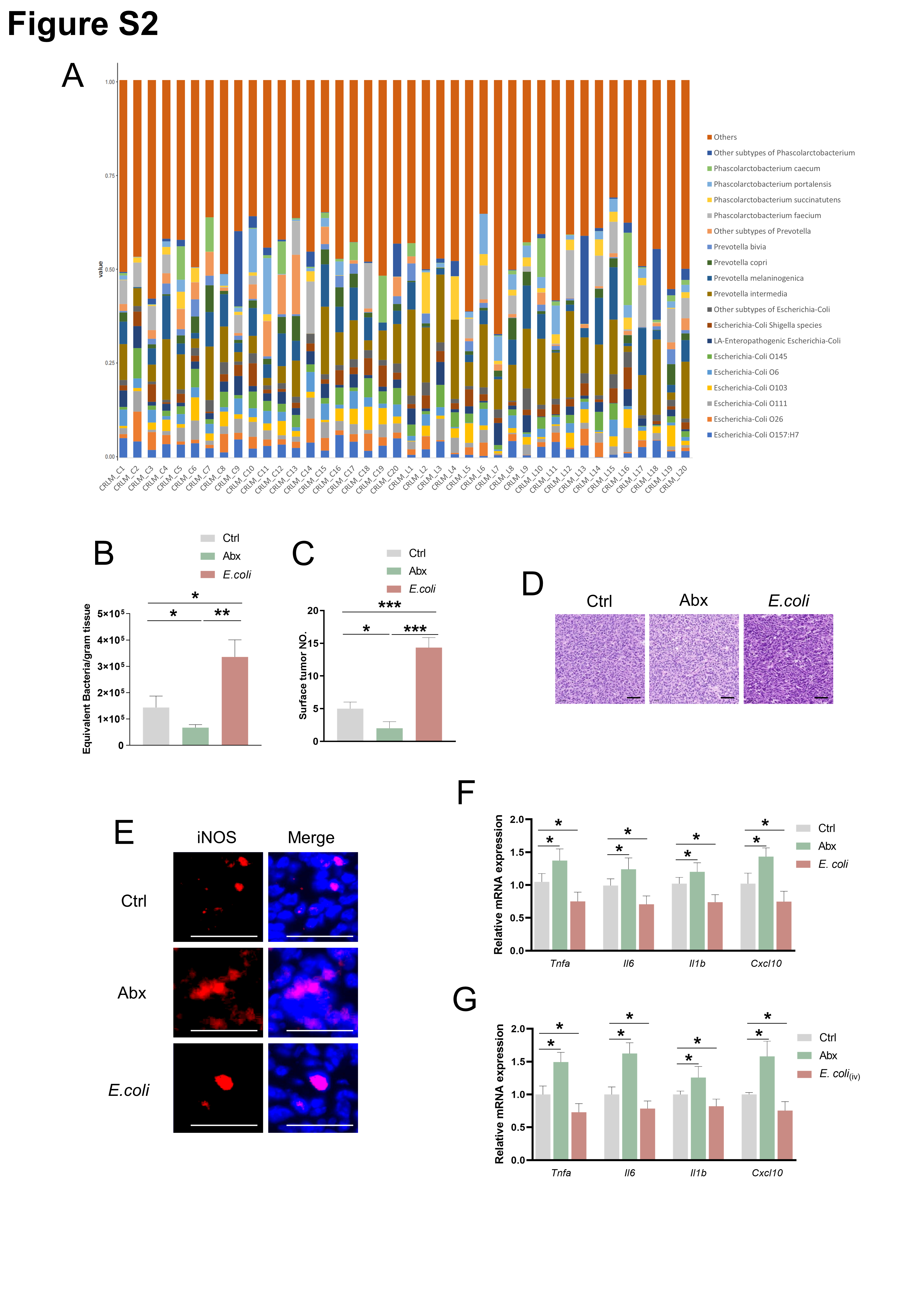

Supplement: Supplementary file 3 — Supplementary Fig. S2 [file 41388_2024_3080_MOESM3_ESM.tif]

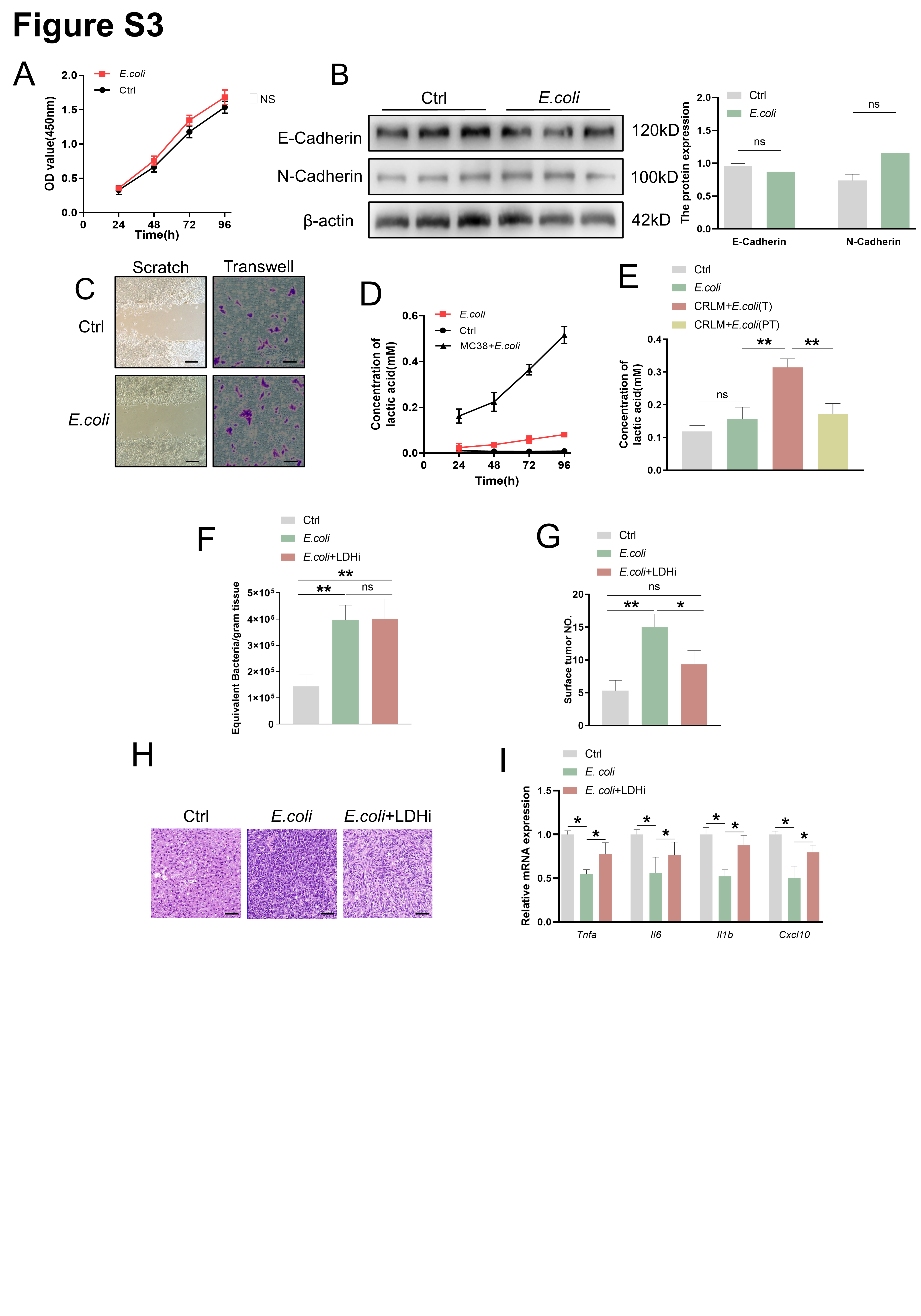

Supplement: Supplementary file 4 — Supplementary Fig. S3 [file 41388_2024_3080_MOESM4_ESM.tif]

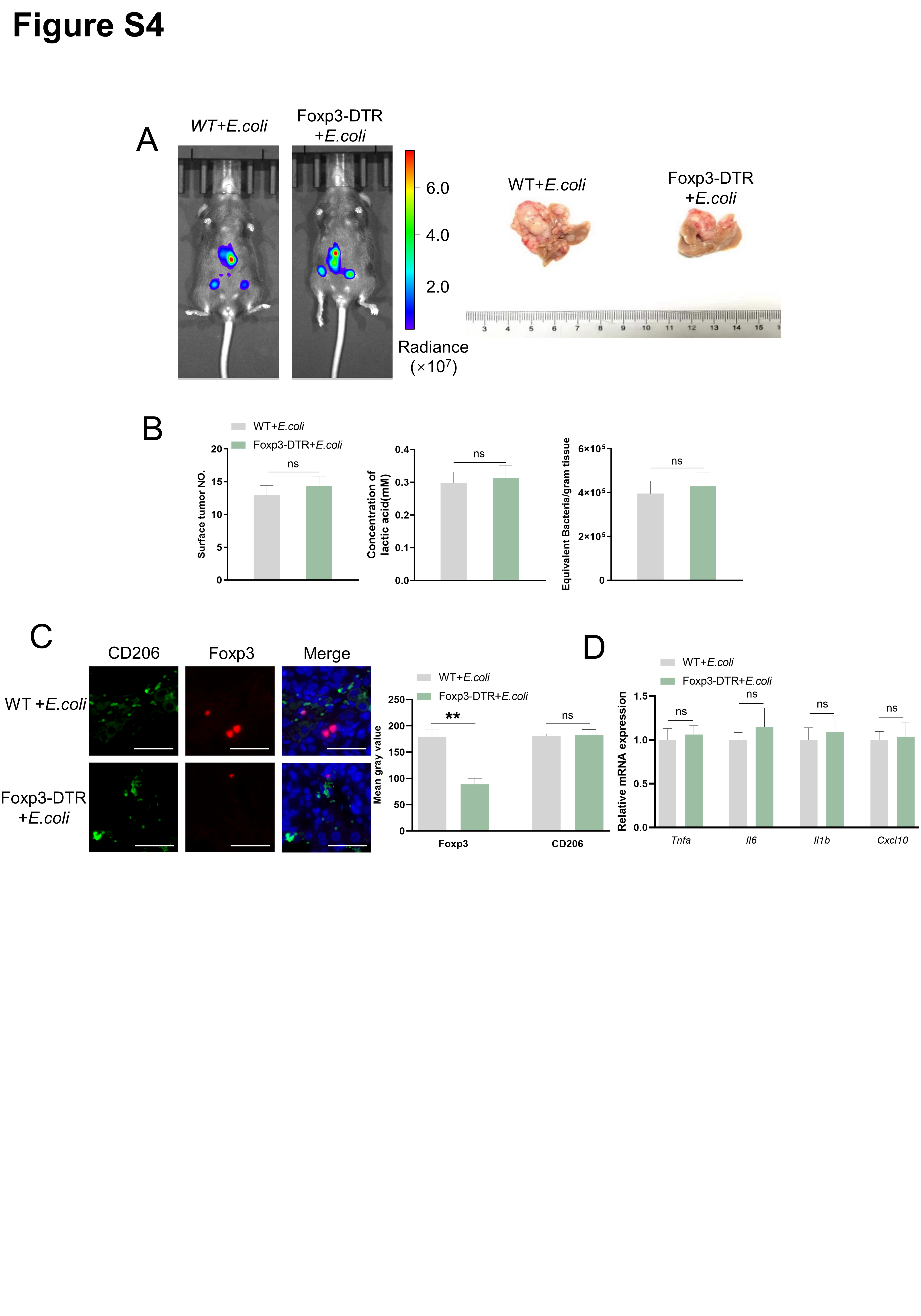

Supplement: Supplementary file 5 — Supplementary Fig. S4 [file 41388_2024_3080_MOESM5_ESM.tif]

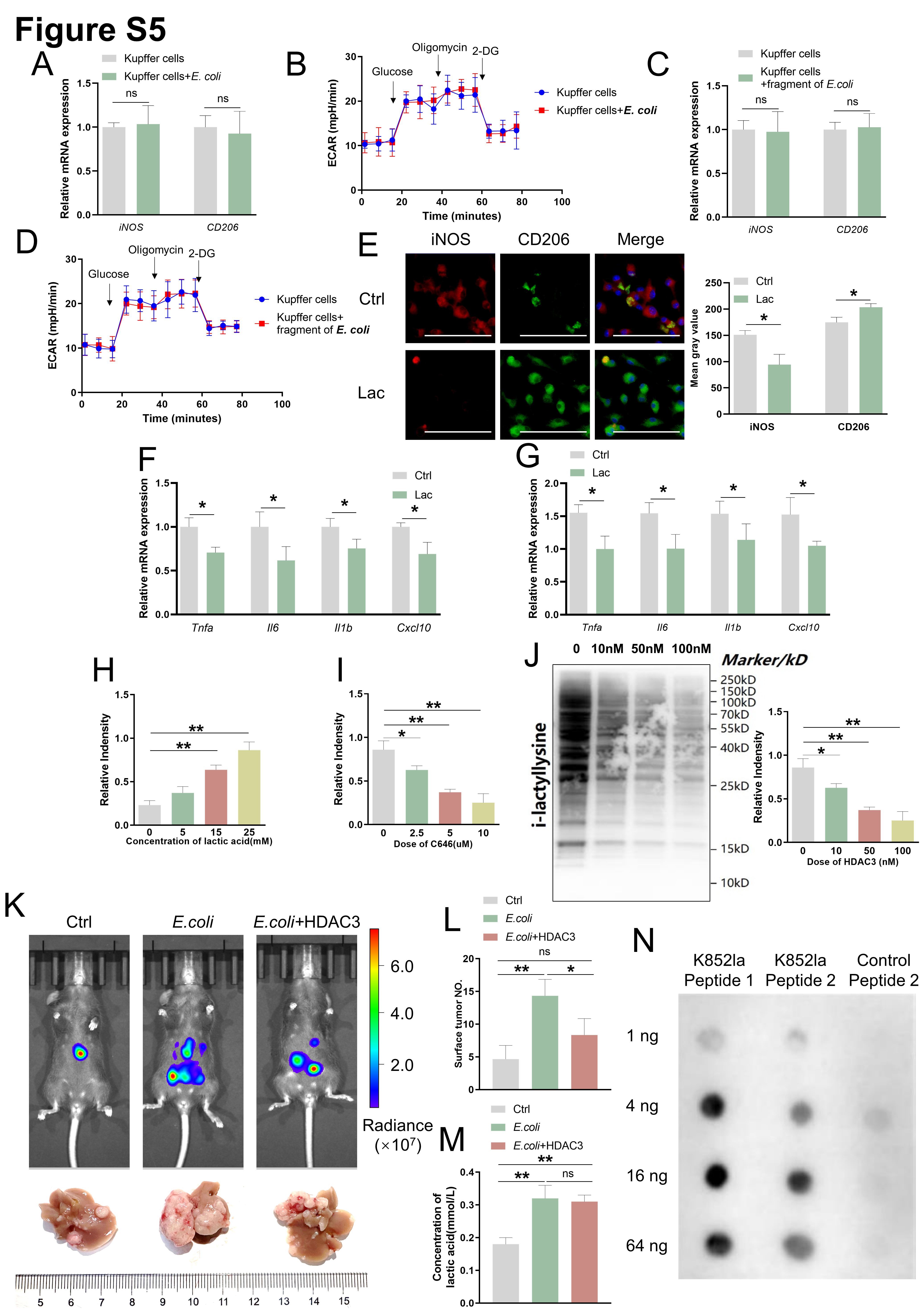

Supplement: Supplementary file 6 — Supplementary Fig. S5 [file 41388_2024_3080_MOESM6_ESM.tif]

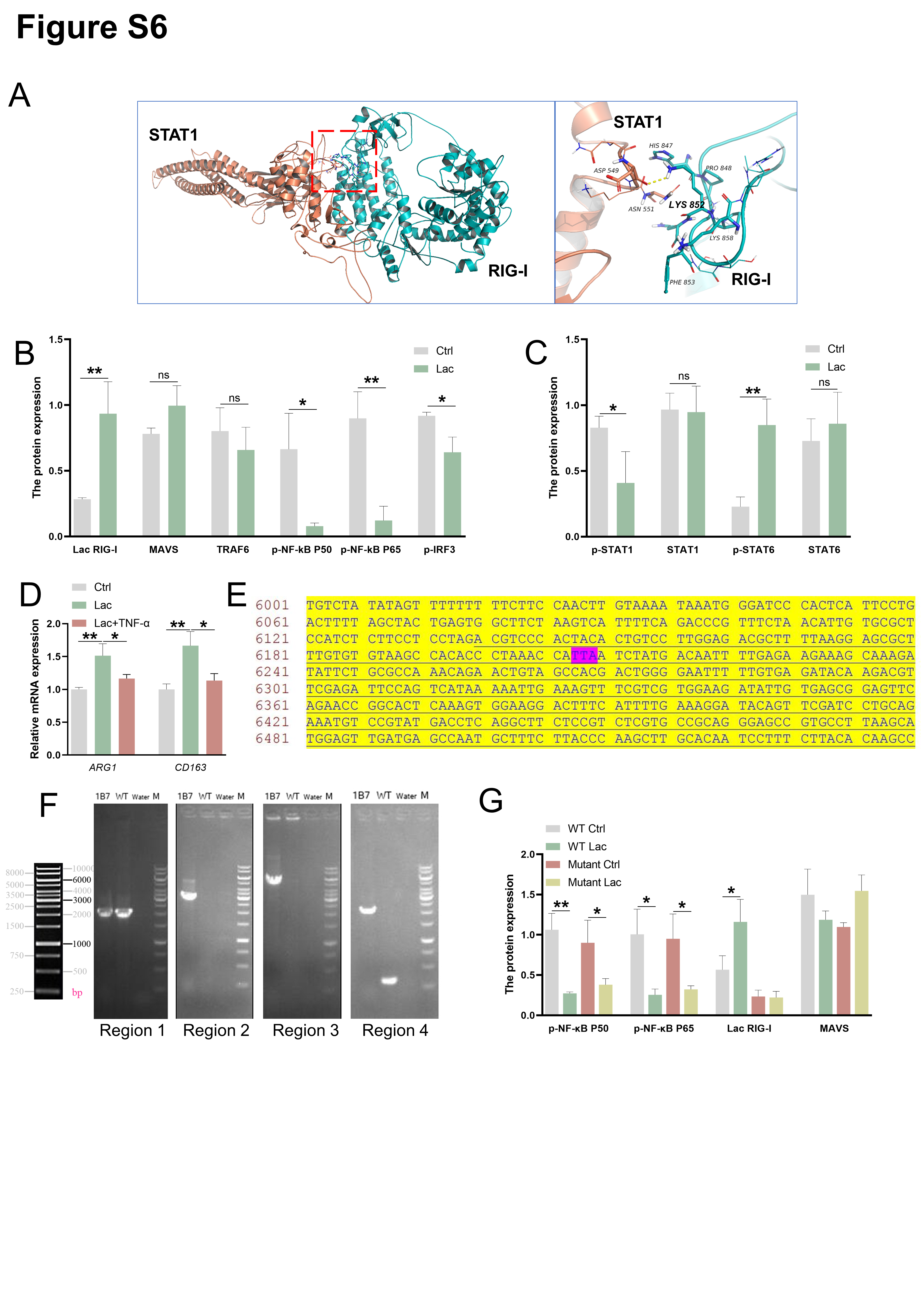

Supplement: Supplementary file 7 — Supplementary Fig. S6 [file 41388_2024_3080_MOESM7_ESM.tif]

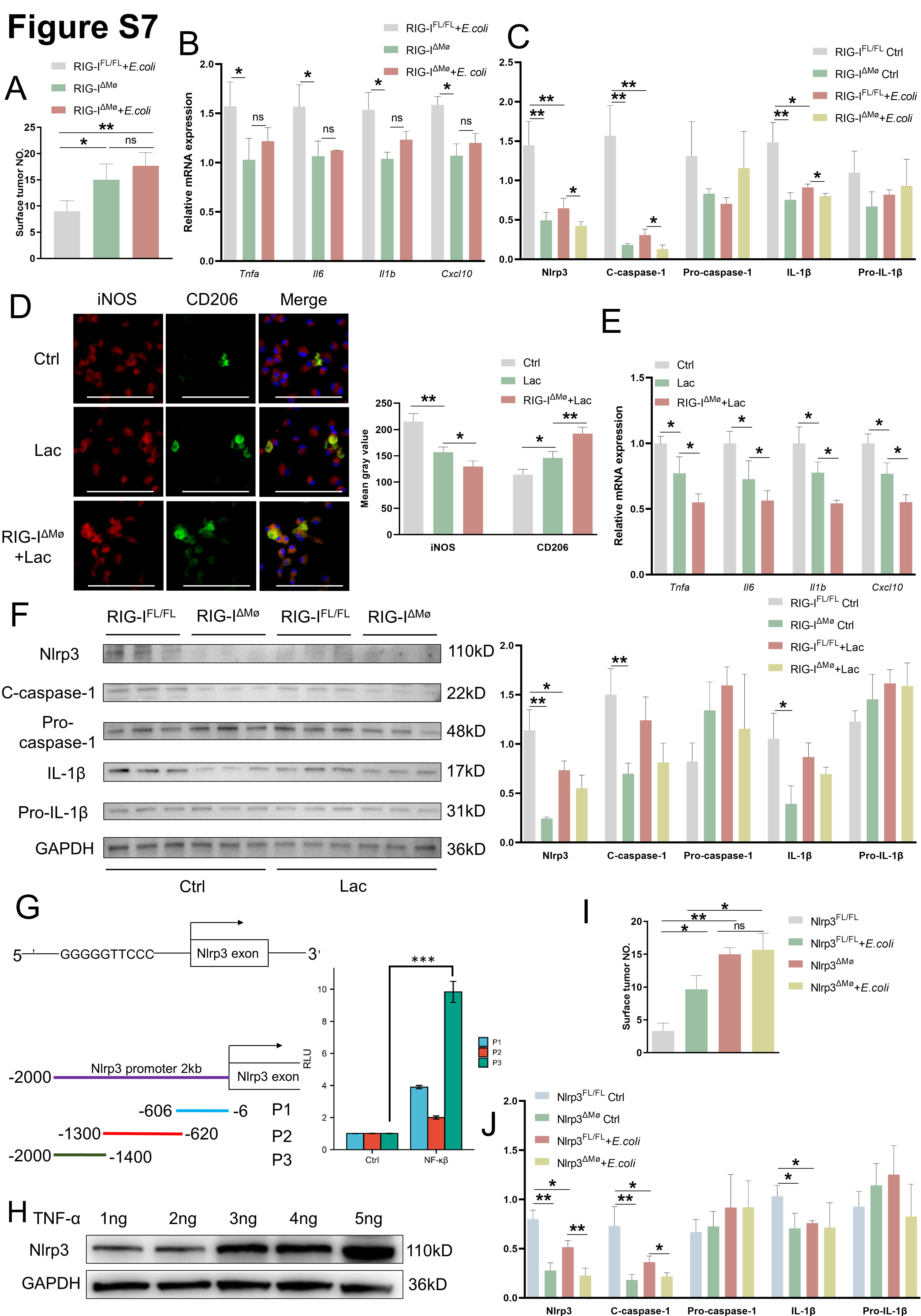

Supplement: Supplementary file 8 — Supplementary Fig. S7 [file 41388_2024_3080_MOESM8_ESM.tif]

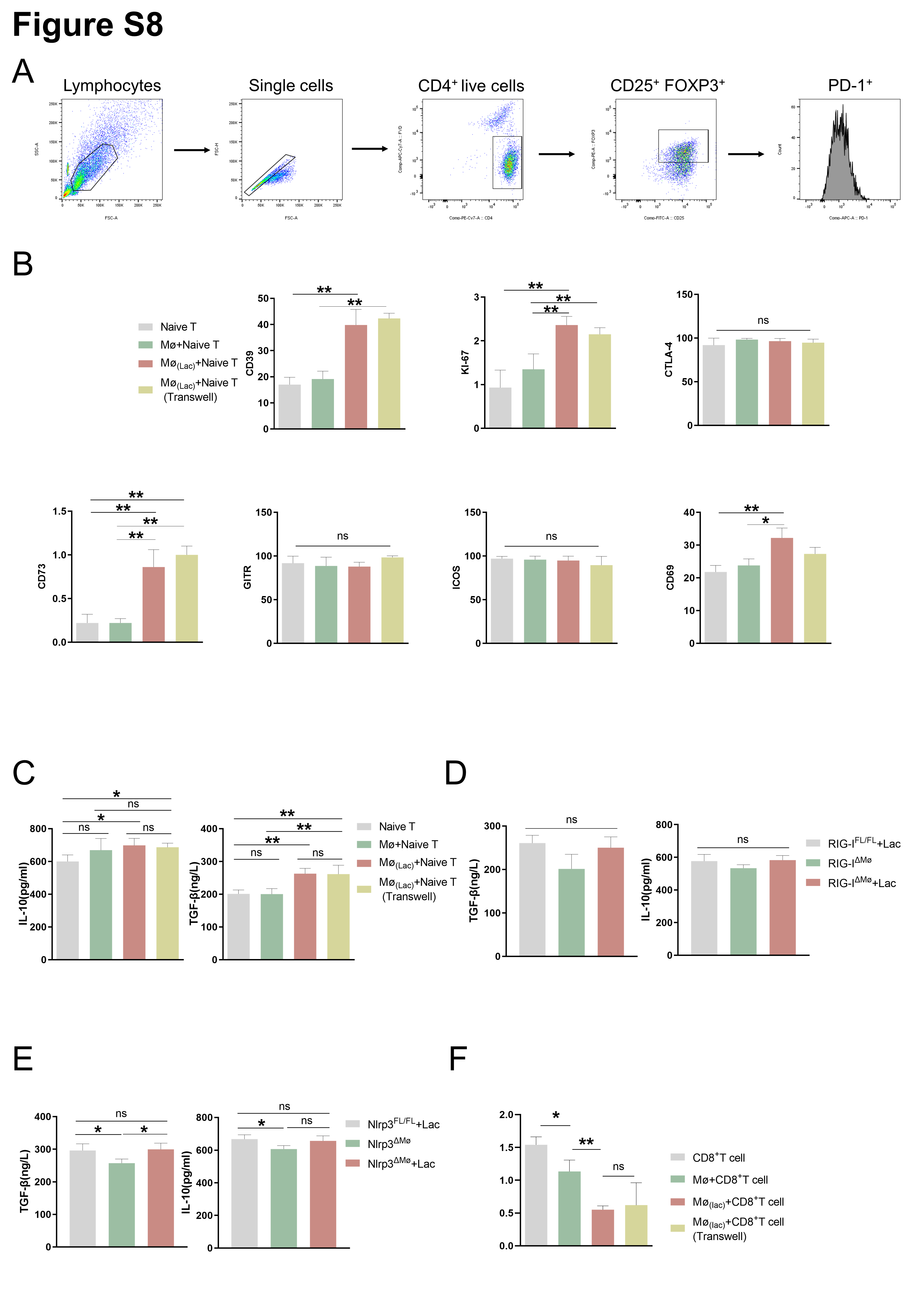

Supplement: Supplementary file 9 — Supplementary Fig. S8 [file 41388_2024_3080_MOESM9_ESM.tif]

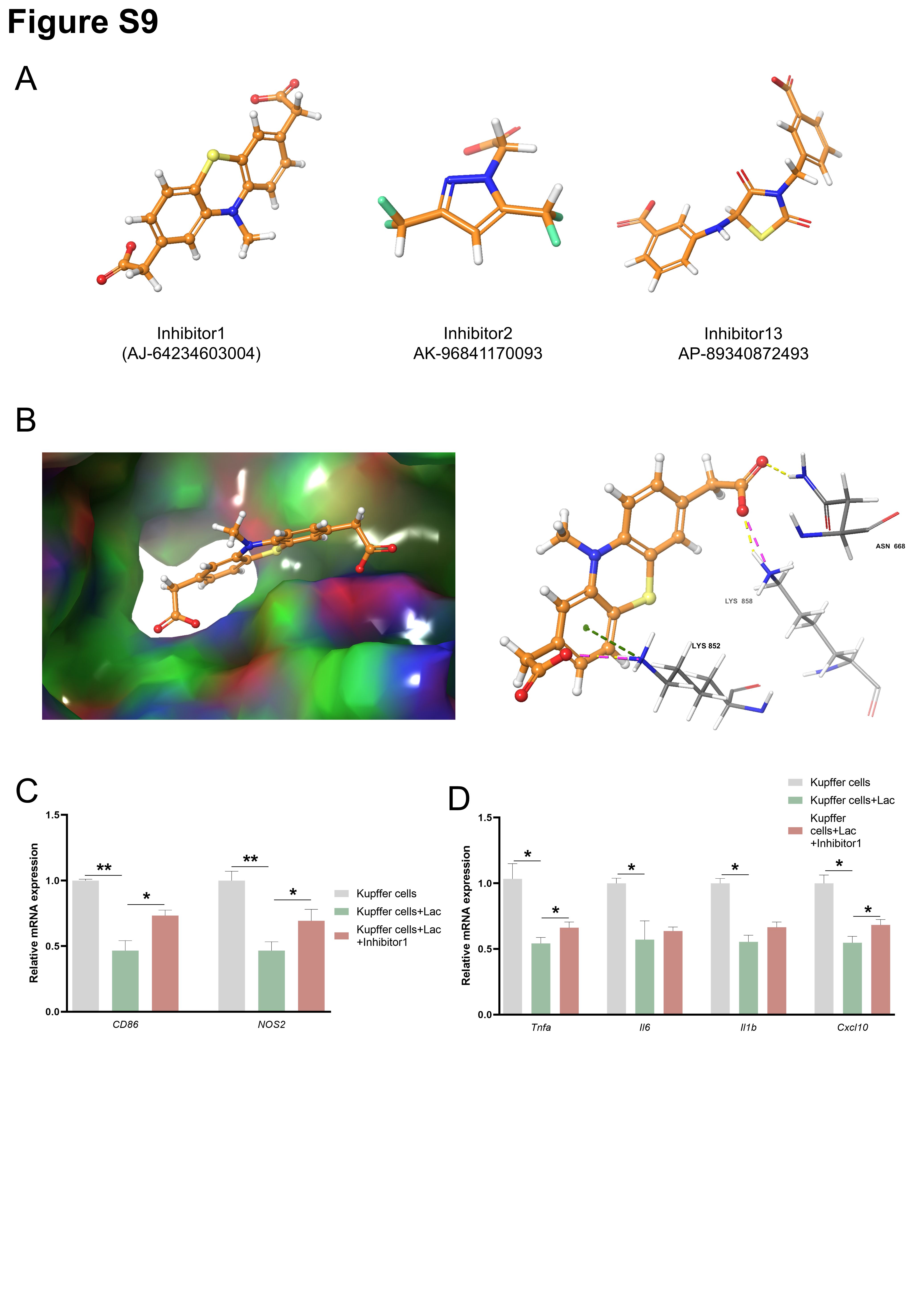

Supplement: Supplementary file 10 — Supplementary Fig. S9 [file 41388_2024_3080_MOESM10_ESM.tif]
